# The ER membrane protein complex promotes biogenesis of sterol-related enzymes maintaining cholesterol homeostasis

**DOI:** 10.1242/jcs.223453

**Published:** 2019-01-16

**Authors:** Norbert Volkmar, Maria-Laetitia Thezenas, Sharon M. Louie, Szymon Juszkiewicz, Daniel K. Nomura, Ramanujan S. Hegde, Benedikt M. Kessler, John C. Christianson

**Affiliations:** 1 Ludwig Institute for Cancer Research, University of Oxford, ORCRB, Headington, Oxford, OX3 7DQ, UK; 2 Target Discovery Institute (TDI) Mass Spectrometry Laboratory, Nuffield Department of Medicine, University of Oxford, Headington, Oxford, OX3 7DQ, UK; 3 Dept. of Chemistry, Molecular and Cell Biology, and Nutritional Sciences and Toxicology, University of California-Berkeley, Berkeley, CA, 94720, USA; 4 MRC Laboratory of Molecular Biology, Francis Crick Avenue, Cambridge CB2 0QH, UK; 5 Oxford Centre for Translational Myeloma Research, NDORMS, University of Oxford, Botnar Research Centre, Headington, Oxford, OX3 7LD, UK

**Keywords:** EMC, Endoplasmic reticulum, Squalene synthase, SOAT1, Cholesterol homeostasis

## Abstract

The eukaryotic endoplasmic reticulum (ER) membrane contains essential complexes that oversee protein biogenesis and lipid metabolism, impacting nearly all aspects of cell physiology. The ER membrane protein complex (EMC) is a newly described transmembrane domain (TMD) insertase linked with various phenotypes, but whose clients and cellular responsibilities remain incompletely understood. We report that EMC deficiency limits the cellular boundaries defining cholesterol tolerance, reflected by diminished viability with limiting or excessive extracellular cholesterol. Lipidomic and proteomic analyses revealed defective biogenesis and concomitant loss of the TMD-containing ER-resident enzymes sterol-O-acyltransferase 1 (SOAT1) and squalene synthase (SQS, also known as FDFT1), which serve strategic roles in the adaptation of cells to changes in cholesterol availability. Insertion of the weakly hydrophobic tail-anchor (TA) of SQS into the ER membrane by the EMC ensures sufficient flux through the sterol biosynthetic pathway while biogenesis of polytopic SOAT1 promoted by the EMC provides cells with the ability to store free cholesterol as inert cholesteryl esters. By facilitating insertion of TMDs that permit essential mammalian sterol-regulating enzymes to mature accurately, the EMC is an important biogenic determinant of cellular robustness to fluctuations in cholesterol availability.

This article has an associated First Person interview with the first author of the paper.

## Abstract (precis)

**Highlighted Article:** The ER membrane protein complex promotes biogenesis of key membrane-bound enzymes responsible for regulation of cholesterol biosynthesis and storage, an important determinant of mammalian cell viability.

## INTRODUCTION

The endoplasmic reticulum (ER) is a diverse organelle whose functions and physical contacts impact nearly all aspects of physiology. Its major roles in the cell include the biogenesis of nearly all secretory and membrane proteins, lipid biosynthesis, Ca^2+^ storage and the regulation of various metabolic pathways. The maintenance of ER homeostasis is therefore of central importance to overall cellular fitness, and identifying the requisite factors and pathways has been a major goal of contemporary cell biology. Accordingly, numerous genetic and biochemical screens have been performed to identify factors that affect ER homeostasis. Large-scale genetic interaction analyses in yeast have identified several ER-resident proteins and complexes with currently unknown functions (Jonikas et al., 2009; Schuldiner et al., 2005). One such complex is the ER membrane protein complex (EMC), an abundant, multi-subunit protein complex, present in all eukaryotic kingdoms (Wideman, 2015).

Yeast EMC was initially described as a stoichiometric complex of six subunits (EMC1–EMC6) whose individual disruption led to activation of the unfolded protein response (UPR) (Jonikas et al., 2009). EMC subunits were subsequently identified in mammalian cells as interactors of known ER-associated degradation (ERAD) components. In mammals, the EMC has ten subunits (EMC1–EMC10) (Christianson et al., 2012), with EMC7 and EMC10 later recognised as Sop4 and YDR056C in yeast (Wideman, 2015). Several studies have identified different EMC subunits in screens for modulators of functional surface expression of membrane proteins, such as the neuronal acetylcholine receptor in *Caenorhabditis elegans* (Richard et al., 2013; Satoh et al., 2015), rhodopsin in *Drosophila* and the ABC transporter Yor1 in yeast (Louie et al., 2012). EMC disruption has also been observed to affect phospholipid trafficking (Janer et al., 2016; Lahiri et al., 2014), autophagosome formation (EMC6, Li et al., 2013; Shen et al., 2016), neurological degeneration (EMC1, Harel et al., 2016), retinal dystrophy (EMC1, Abu-Safieh et al., 2013), SV40 egress from the ER (EMC1, Bagchi et al., 2016), and pathogenesis of flaviviruses including West Nile, Dengue and Zika (Le Sommer et al., 2012; Ma et al., 2015; Marceau et al., 2016; Savidis et al., 2016; Zhang et al., 2016). The function(s) of the EMC linking these diverse phenotypes across various organisms remain an area of active investigation. In recent advances, the EMC was shown to be able to serve as an insertase for weakly hydrophobic transmembrane domains of tail-anchored (TA) proteins (Guna et al., 2018), modulate the co-translational expression of multi-pass membrane proteins with challenging TMDs (Shurtleff et al., 2018) and promote accuracy of G-protein-coupled receptor (GPCR) biogenesis through insertion of their first TMD (Chitwood et al., 2018). How the insertase activity of EMC underlies the range of phenotypes reported is not yet clear.

Here, we determine fundamental aspects of EMC assembly and architecture in mammalian cells. Leveraging these insights revealed that cells lacking the EMC are sensitive to extracellular cholesterol availability. By undertaking lipidomic analyses and quantitative proteomics, we identified lipid species and proteins whose abundance was dependent on the EMC, including multiple factors intimately tied to cholesterol homeostatic maintenance. Biochemical and cell biological analyses demonstrated that the loss of these essential factors was due to premature degradation, implicating the EMC in assuring their correct biogenesis. We propose that robust maintenance of cholesterol homeostasis requires the insertase activity of the EMC for the optimal integration of essential biosynthetic and storage enzymes into the ER membrane. This function, and the immediate consequences for lipid and protein homeostasis, likely contribute to the diverse cellular and organismal phenotypes caused by loss of the EMC.

## RESULTS

### EMC integrity is maintained by a set of essential subunits

**Fig. 1.**
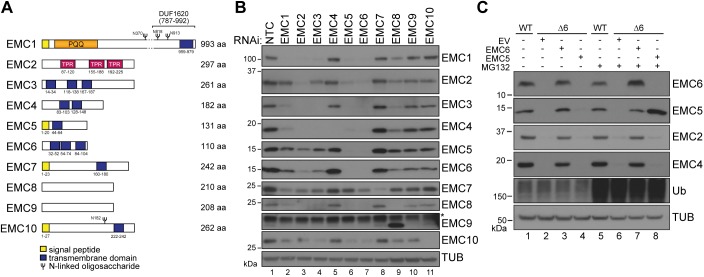
**EMC5 and EMC6 are essential for EMC maturation.** (A) Schematic representation of the primary structure of all EMC subunits (EMC1–EMC10). Domains, boundary residue numbers and predicted glycosylation sites are indicated. Pyrrolo-quinoline quinone (PQQ) and tetratricopeptide repeats (TPR) are shown. (B) siRNA-mediated depletion of EMC1–EMC10 and non-targeting control (NTC) for 72 h in U2OS Flp-In™ T-Rex™ cells. Whole-cell lysates (WCL) of individually depleted cells were separated by SDS-PAGE and resulting western blots probed for each subunit and tubulin (TUB) as indicated. The asterisk (*) denotes a nonspecific band. (C) U2OS Flp-In™ T-Rex™ cells modified by CRISPR/Cas9 to knockout EMC6 (Δ6) were reconstituted by inducing expression of an empty vector control (EV), EMC5 or EMC6 (DOX 1 ng/ml, 72 h). MG132 was added to cells where indicated (5 µg/ml, 8 h). Samples were prepared as in B. TUB, tubulin; Ub, ubiquitin.

The mammalian EMC contains ten distinct subunits (Christianson et al., 2012) that differ extensively in both primary sequence and membrane topology (Fig. 1A). To rationally target the EMC in functional studies, we first sought to understand how each subunit contributes to the integrity of the mature complex. We monitored stability of the complex in response to subunit knockdown. All subunits of the EMC shown previously to co-purify (Guna et al., 2018), were observed to co-sediment as a single complex on sucrose gradients (Fig. S1A, fractions 7–9). Individually silencing EMC1, 2, 3, 5 or 6 by means of siRNAs or sgRNAs caused marked co-depletion of the remaining EMC subunits, whereas depletion of EMC4, 7, 9 or 10 was not notably disruptive (Fig. 1B; Fig. S1B,C). EMC8 knockdown reduced the levels of some subunits, but led to an increase in EMC9 (Fig. 1B, lane 9). The similarity of EMC8 and EMC9 (>40% amino acid identity) suggests that EMC9 might partially compensate for EMC8 loss. Although almost all EMC subunits were lost in EMC6 knockdowns, their corresponding mRNA levels were not significantly changed (Fig. S1D), suggesting that the remaining subunits are degraded post-translationally. As expected, any remaining EMC subunits in these knockdown experiments showed altered sedimentation profiles (Fig. S1C), illustrating that the intact complex was disrupted.

As EMC integrity was most severely disrupted when EMC5 or EMC6 were depleted, we focused on these subunits for subsequent studies. Because of their importance to assembly, EMC5 and EMC6, along with EMC2 and EMC3 were designated as ‘core’ subunits of the EMC. We genetically modified U2OS Flp-In™ T-Rex™ cells by CRISPR/Cas-9 to generate knockouts of EMC5 (Δ5) or EMC6 (Δ6) (Fig. 1C; Fig. S1E–G, and see Materials and Methods), then stably reintroduced the respective genes into the single tetracycline-inducible FRT recombination locus. As with the knockdown experiments, most EMC subunits were at nearly undetectable levels in both the ΔEMC5 and ΔEMC6 cells (hereafter collectively referred to as ΔEMC). Doxycycline (DOX) induction restored the missing subunit and consequently the EMC to levels near normal (Fig. 1C). The finding that EMC5 expressed in Δ6 cells can be stabilised by including the proteasome inhibitor MG132 is evidence that co-depletion of EMC subunits occurs post-translationally (Fig. 1C, compare lanes 4 and 8). Furthermore, it suggests that EMC6 is the core subunit that initiates assembly of the EMC. These cell lines provided the means to systematically investigate the role(s) of EMC in mammalian cell physiology.

### EMC deficiency sensitises cells to cholesterol excess and starvation

**Fig. 2.**
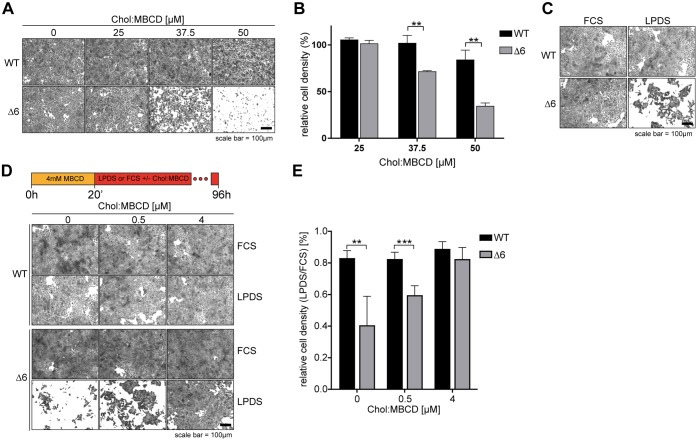
**EMC-deficient cells are sensitised to cholesterol surplus and starvation.** (A) WT and ΔEMC6 (Δ6) cells were exposed to Chol:MBCD (25, 37.5 and 50 µM, 20 h) and visualised by staining with Crystal Violet. (B) Quantification of cell densities for experiments as in A normalised to untreated cells (0 µM Chol:MBCD). Means±s.d. are shown (*n*=3). **P≤0.01 (Student's *t*-test). (C) WT and Δ6 cells depleted of cholesterol by MBCD (4 mM, 20 min) were switched to FCS- (5%) or LPDS- (5%) containing growth media (96 h) and visualised by staining with Crystal Violet. (D) Cells were treated as in C with or without Chol:MBCD (0.5 or 4 µM). (E) Quantification of cell density from experiments shown in D. Means±s.d. are shown (*n*=4). **P≤0.01, ***P≤0.001 (Student's *t*-test).

Disruptions to individual EMC subunits in both cell lines and model organisms have produced a wide range of seemingly unrelated phenotypes (Bagchi et al., 2016; Brockerhoff et al., 1997; Jonikas et al., 2009; Lahiri et al., 2014; Ma et al., 2015; Richard et al., 2013; Tang et al., 2017). Because the EMC6 knockout produced a near-complete loss of the complex, we screened ΔEMC6 cells for viability under a set of growth conditions. During this, we observed ΔEMC6 cell lines to be particularly sensitive to the amount of extracellular cholesterol available from growth media. EMC-deficient cells grew as well as wild-type (WT) cells under standard growth conditions (DMEM plus 10% FCS), but showed significantly diminished viability in media supplemented with cholesterol delivered in complex with the binding agent methyl-β-cyclodextrin (Chol:MBCD) (Fig. 2A,B). Remarkably, limiting extracellular cholesterol availability also strongly reduced the viability of ΔEMC6 cells but not of WT cells. This cholesterol auxotroph phenotype was seen when the cells were grown in lipoprotein-depleted serum (LPDS, Fig. 2C) or with uncomplexed MBCD (Fig. S2A,B). Restoring EMC6 expression to ΔEMC6 cells (Δ6+6) reversed the sensitivity to cholesterol depletion, rescuing viability to WT levels (Fig. S2A–C) and ruling out effects unrelated to EMC loss. Importantly, we determined that a minimal non-toxic amount of Chol:MBCD (4 µM) restored viability to ΔEMC6 cells (Fig. 2D,E), confirming it is excess cholesterol that EMC-deficient cells are unable to tolerate.

### Cholesterol homeostatic responses are intact in EMC-deficient cells

**Fig. 3.**
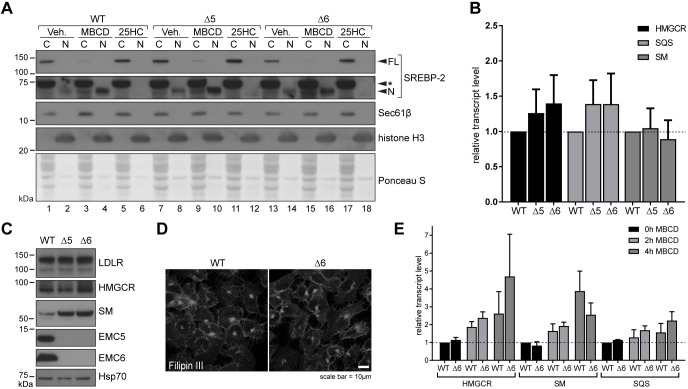
**EMC loss does not activate a cholesterol homeostatic response.** (A) Isolation of cytoplasmic (C) and nuclear (N) fractions from WT and ΔEMC6 (Δ6) cells by subcellular fractionation. Cells were treated with MBCD (1 mM), 25-hydroxycholesterol (25HC, 25 µM) or ethanol (control, Veh.) for 4 h. Western blots probing for the full-length (FL) and cleaved N-terminal fragment (N) of SREBP-2 are shown, along with Sec61β and histone H3 as controls for cytoplasmic and nuclear fractions, respectively. The asterisk (*) denotes a nonspecific band. (B) mRNA levels of HMGCR, SQS and SM as determined by quantitative real-time RT-PCR (qRT-PCR). Transcript levels present in untreated ΔEMC5 (Δ5) and Δ6 U2OS Flp-In™ T-Rex™ cells relative to WT are shown as means±s.d. (*n*=6). (C) Western blots of whole-cell lysates derived from WT, Δ5 and Δ6 cells grown in normal medium were probed with the indicated antibodies, where Hsp70 served as a loading control. (D) Filipin III staining of WT and Δ6 cells maintained in DMEM plus 10% FCS to monitor intracellular cholesterol distribution. (E) mRNA levels of HMGCR, SM and SQS from WT and Δ6 cells treated with 1 mM MBCD for 0, 2 and 4 h, as determined by qRT-PCR relative to WT. Means±s.d. are shown (*n*=3).

Failing to adapt to changes in exogenous cholesterol availability suggested that cholesterol homeostatic mechanisms might be compromised in ΔEMC cells. The ER-resident transcription factor sterol regulatory element-binding protein-2 (SREBP-2, also known as SREBF2) exerts homeostatic control over free cholesterol levels in cells by rapidly sensing cholesterol insufficiency. Relocation of SREBP-2 to the Golgi and release of its N-terminus [SREBP-2(N)] owing to the action of proteases, allows the active transcription factor to enter the nucleus and activate cholesterogenic genes (Adams et al., 2004; Brown et al., 2002). Surprisingly, the amount of SREBP-2(N) detected in nuclear fractions did not differ markedly between WT, Δ5 and Δ6 cell lines when grown in standard medium (Fig. 3A, compare lanes 2, 8 and 14), indicating that SREBP-2 did not sense any substantial disruption to free cholesterol levels in EMC-knockout cells. Cholesterol depletion with sub-lethal amounts of MBCD led to efficient SREBP-2 cleavage in each of the cell lines (Fig. 3A, compare lanes 4, 10 and 16), whereas addition of 25-hydroxycholesterol (25-HC), which promotes ER retention of SREBP-2, abolished even the small basal cleavage observed under normal conditions (Fig. 3A, compare lanes 6, 12 and 18). These findings indicate that SREBP-2 activity and responsiveness are normal in ΔEMC cells but not activated.

Transcription of representative SREBP-2 target genes, whose activation reinforces cholesterol biosynthesis and uptake to restore homeostasis, was slightly elevated in ΔEMC6 cells (Fig. 3B), but failed to reach statistical significance. Immunoblotting (Fig. 3C) and RNA-Seq analyses (Fig. S3) of ΔEMC cells supported these data, revealing no evidence of coordinated upregulation within genes of the cholesterol biosynthetic pathway at steady state. One exception was the elevation of squalene monooxygenase (SM, also known as SQLE; EC 1.14.14.17) at the protein (Fig. 3C) but not the mRNA level (Fig. 3B), which may be linked to sterol-dependent feedback controlling its degradation by the ubiquitin ligase MARCH6 (the equivalent of yeast Doa10) (Foresti et al., 2013; Gill et al., 2011). Cellular cholesterol was not differentially distributed in ΔEMC6 and WT cells (Fig. 3D). Furthermore, the transcriptional response of SREBP2-dependent genes [HMGCR, SM and SQS (also known as FDFT1)] to acute cholesterol depletion in ΔEMC6 cells was comparable to that in WT cells (Fig. 3E). Even though ΔEMC cells are intolerant of exogenous cholesterol extremes, they still appear to maintain adequate free cholesterol levels under standard growth conditions. Thus, the sensitivity of EMC-deficient cells to extracellular cholesterol fluctuations cannot be explained by aberrant homeostatic pathways.

### EMC deficiency compromises cholesterol storage

**Fig. 4.**
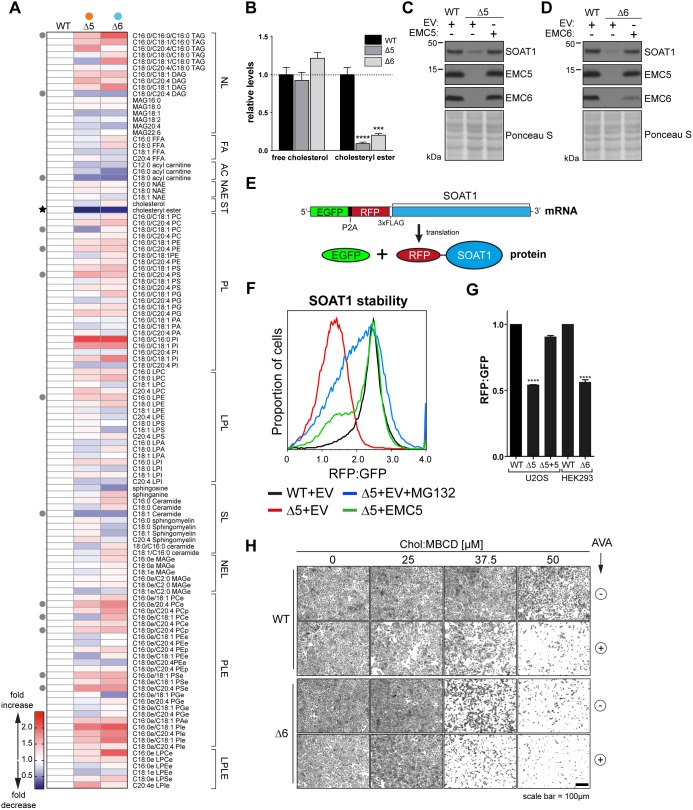
**SOAT1 loss from EMC-deficient cells and attenuated cholesteryl ester formation.** (A) Heat map representing the fold change of 117 lipid species in EMC5 (Δ5 #5-4) and EMC6 (Δ6 #3-9) deletion mutants relative to WT as identified by targeted metabolomic analysis (*n*≥4). Lipid species are arranged according to major structural class. The 14 lipid species significantly altered in both Δ5 and Δ6 cells (*P*≤0.05) are indicated (grey circles, black star). NL, neutral lipids; FA, fatty acids; AC, acyl carnitine; NAE, N-acylethanolamines; ST, sterols; PL, phospholipids; LPL, lysophospholipids; SL, sphingolipids; NEL, neutral ether lipids; PLE, phospholipid ethers; LPLE, lysophospholipid ethers. (B) Quantification of free cholesterol and cholesteryl esters in Δ5 and Δ6 cells relative to WT (dashed line). Means±s.e.m. are shown (*n*=4). ***P≤0.001, ****P≤0.0001 (Student's *t*-test). (C,D) Western blots of whole-cell lysates (WCL) for Δ5 cells with or without the EMC5 expression vector (C) and Δ6 cells with or without the EMC6 expression vector (D) probed for SOAT1 and indicated EMC subunits. (E) Schematic representation of the dual reporter construct used in F–G. mRNA encoding GFP separated by a P2A sequence from RFP and FLAG-tagged SOAT1. Translation results in ribosome skipping and the generation of GFP and RFP-3xFLAG-SOAT1 at equimolar ratios. Differences in stability between both gene products gives rise to altered RFP:GFP ratios, serving as a sensitive readout for protein stability. (F) Fluorocytometric RFP:GFP ratio in WT and Δ5 cells with or without the EMC5 expression vector and transiently expressing GFP-P2A-RFP-3xFLAG-SOAT1. At 24 h post transfection, cells were treated with MG132 (5 µg/ml) or DMSO (vehicle control) for 8 h and analysed by flow cytometry. EV, empty vector control. (G) Quantification of three independent experiments as performed in F. Means±s.d. are shown (*n*=3). ****P≤0.0001 (Student's *t*-test). (H) WT and Δ6 cells were exposed to Chol:MBCD (25, 37.5 and 50 µM, 20 h) with or without 10 µM avasimibe (AVA, 20 h) and visualised by staining with Crystal Violet.

During efforts to explore the basis of the ΔEMC phenotype, we performed whole-cell lipidomic analysis to identify potential points of dysregulation (Fig. 4A). We considered only lipid species that were consistently and significantly altered (*P*≤0.05) in both ΔEMC5 and ΔEMC6 cells to minimise any clonal bias. The most striking effect was a ∼10-fold and ∼5-fold decrease in cholesteryl esters in both ΔEMC5 and ΔEMC6 cells (Fig. 4A,B), while most other lipid species (fatty acids, neutral lipids, sphingolipids etc.), including cholesterol, appeared relatively unaffected (Fig. S4A–J). Among those lipids affected by EMC loss, the changes in most did not reach statistical significance. Based on these findings, we focused on cholesteryl esters because they represent the principal form of stored cholesterol in mammalian cells (Chang et al., 2009) and are likely to be connected to the cholesterol sensitivity seen in ΔEMC cells.

### SOAT1 expression is EMC-dependent

Cholesteryl esters are produced by sterol-O-acyltransferase/acyl-CoA-acetyltransferase 1 (SOAT1, also known as ACAT1; EC 2.3.1.26), a homotetrameric ER-resident enzyme (Chang et al., 2009). SOAT1 uses long chain fatty acids to modify surplus free cholesterol for inert storage in lipid droplets (LDs) of extrahepatic tissues (Pol et al., 2014). In hepatic and gastrointestinal tissues, this role is comparably performed by the SOAT1 homolog (46% identity), SOAT2 (also known as ACAT2) (Oelkers et al., 1998). Cholesterol esterification by SOAT1 represents an important mechanism to control free cholesterol levels in cells and reduce the potential cytotoxicity associated with its accumulation (Feng et al., 2003; Warner et al., 1995). SOAT1 protein was markedly reduced in both EMC5 and EMC6 knockouts across multiple cell lines (Fig. 4C,D; Fig. S4K,L), while transcript levels did not differ significantly from WT (Fig. S4M). Importantly, SOAT1 returned to WT levels by restoring the EMC with the missing subunit (Δ5+EMC5, Δ6+EMC6; Fig. 4C,D).

To confirm the SOAT1 loss in EMC-deficient cells was post-transcriptional, we constructed a dual fluorescence reporter using GFP-(P2A)-RFP fused to SOAT1. When translated, a ribosome-skipping event induced by the P2A site releases the N-terminal GFP from RFP–SOAT1 at equimolar ratios (Fig. 4E; Fig. S4N). Conditions favouring RFP–SOAT1 destabilisation relative to GFP decrease the RFP:GFP ratio, in effect serving as a quantitative readout for protein stability (Guna et al., 2018). Transient expression of the SOAT1 reporter in EMC-deficient cells exhibited marked reduction of RFP:GFP ratios compared to expression in WT (Fig. 4F,G). Rescuing ΔEMC5 cells by expressing EMC5 (Δ5+EMC5) or treating with the proteasome inhibitor MG132 restored SOAT1 to levels near to those found in WT (Fig. 4F,G).

To assess whether SOAT1 destabilisation contributed to the heightened sensitivity of ΔEMC6 cells to elevated cholesterol (Fig. 2A), we treated WT and EMC-deficient cells with the SOAT1 inhibitor avasimibe (AVA) under conditions of high cholesterol load (+Chol:MBCD). WT cells were sensitive to cholesterol loading only in the presence of AVA, comparable to that observed for untreated ΔEMC6 cells (Fig. 4H). Of note, AVA further enhanced sensitivity of ΔEMC6 cells to Chol:MBCD-induced cell death, indicating that SOAT1 activity might be limiting but not entirely absent. These findings indicate a post-transcriptional defect in SOAT1 biogenesis coinciding with EMC deficiency that results in degradation and compromised esterification capacity.

### Various intracellular proteins exhibit EMC dependence

**Fig. 5.**
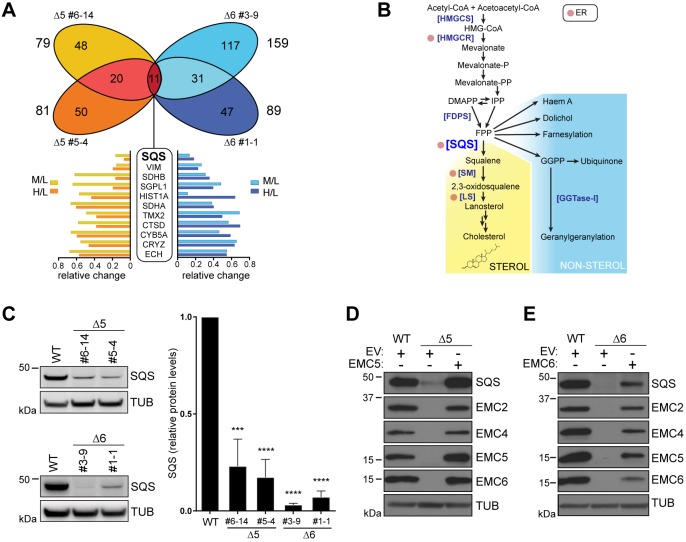
**Loss of SQS expression from EMC-deficient cells.** (A) Venn diagram representing proteins decreased by ≥30% in ΔEMC5 and ΔEMC6 cells in triple-label SILAC assays. Relative protein ratios of ΔEMC5 (Δ5 #5-4, Δ5 #6-14) and ΔEMC6 (Δ6 #1-1, Δ6 #3-9) U2OS Flp-In™ T-Rex™ cells labelled with deuterated heavy (H) or medium (M) amino acids were compared to WT cells (L) by tandem mass spectrometry. Proteins decreased by ≥30% in all knockout cell lines are indicated (boxed; gene names shown) with SQS highlighted (bold). The relative change of each candidate's signature peptides (M/L, H/L) is indicated below (bar graphs). (B) Schematic representation of the sterol and non-sterol isoprenoid biosynthesis pathways. ER-resident enzymes are indicated (red circle). Abbreviations from top to bottom: HMGCS, HMG-CoA synthase; HMGCR, HMG-CoA reductase; DMAPP, dimethylallyl pyrophosphate; IPP, isopentenyl pyrophosphate; FPP, farnesyl pyrophosphate; FDPS, FPP synthase; SQS, squalene synthase; SM, squalene monooxygenase; LS, lanosterol synthase; GGTase-I, geranylgeranyl transferase type I. (C) Validation of SQS loss in EMC-deficient cells. Left: western blots of whole-cell lysates (WCLs) from ΔEMC5 (Δ5) and ΔEMC6 (Δ6) cells used in A and probed for SQS. Right, densitometric analysis of SQS relative abundance normalised to WT. Means±s.d. are shown (*n*=3). ***P≤0.001, ****P≤0.0001 (Student's *t*-test). Reconstitution of Δ5 (D) and Δ6 cells (E) with empty vector control (EV), EMC5 or EMC6. Western blots of WCLs were probed with antibodies for SQS, EMC2, EMC4, EMC, EMC5, EMC6 and tubulin (TUB).

While loss of SOAT1 clarified the observed sensitivity to surplus extracellular cholesterol, it could not readily explain the cholesterol auxotropy of ΔEMC cells. To investigate this, we used stable isotope labelling with amino acids in cell culture (SILAC) and quantitative proteomic analysis to compare WT and ΔEMC cells (Fig. S5A). By adopting stringent selection criteria that required reduction by ≥30% in two independent ΔEMC5 and ΔEMC6 cell lines, we aimed to mitigate any bias arising from clonal variation. Analysis of total intracellular proteins identified 11 unique proteins whose signature peptides met these criteria (Fig. 5A; Fig. S5B,C). Of the proteins identified, SQS (EC 2.5.1.21), was the one consistently and most substantially downregulated across all four cell lines. This marked effect, together with its central position in the cholesterol biosynthesis pathway, led us to investigate SQS further.

### SQS and cholesterol auxotrophy in EMC-deficient cells

SQS is an evolutionarily conserved ER-resident enzyme catalysing the first committed step in *de novo* sterol production (Beytía and Porter, 1976; Do et al., 2009). SQS reductively condenses two farnesyl-pyrophosphate (FPP) molecules to form the linear triterpene intermediate squalene (Fig. 5B; Ye and DeBose-Boyd, 2011). It is positioned at a critical branch point of metabolism, determining flux of the mevalonate pathway product FPP into sterol and non-sterol isoprenoid-dependent processes (Espenshade and Hughes, 2007). Importantly, cells lacking SQS (ΔSQS) or treated with the SQS inhibitor zaragozic acid (ZA) (Bergstrom et al., 1993) were acutely sensitive to cholesterol depletion-induced cell death (by MBCD or LPDS, Fig. S5D,E), mimicking the sensitivity shown by ΔEMC6 cells to identical conditions (Fig. 2C, Fig. S2A). Without SQS activity, the FPP precursor cannot supply the sterol biosynthetic pathway and instead is shifted towards the production of isoprenoids. Both ΔEMC6 and ZA-treated WT cells diverted more FPP into the non-sterol isoprenoid pathway, reflected by the increase in a geranylgeranylated form of Rap1a (Fig. S5F) as determined by using an antibody specific for unprenylated Rap1a (Leichner et al., 2011). This is consistent with an attenuated SQS activity that impairs sterol biosynthesis. Thus, the loss of SQS from EMC-deficient cells could explain the auxotrophic behaviour of these cells for cholesterol.

Consistent with the SILAC data, we found significantly lower SQS in each EMC-deficient cell line (ΔEMC5/6) by both western blotting (Fig. 5C) and indirect immunofluorescence (Fig. S5G). Loss of SQS was also observed in A673 and HEK293 cell lines (Fig. S5H,I), illustrating a fundamental dependency of SQS expression on the EMC. Reminiscent of what was observed for SOAT1, restoring the fully assembled EMC with EMC5 (Δ5+EMC5) or EMC6 (Δ6+EMC6) returned SQS expression to near WT levels (Fig. 5D,E). Depleting selected EMC subunits (e.g. EMC2–EMC6) but not others (EMC1, EMC7–EMC10) also prevented SQS maturation (Fig. S5J,K), indicating that, despite being part of a shared complex, not all subunits may be contributing to its function. Thus, a fully functional EMC is essential for maintaining the steady-state levels of the cholesterogenic enzyme SQS and the cholesterol storage enzyme SOAT1.

### SQS and SOAT1 expression defects are independent

Given that SQS and SOAT1 both exert control over cholesterol availability within the cell, their relative abundance may be interdependent. However, the loss of SQS activity (ΔSQS, +ZA) did not affect SOAT1 expression levels (Fig. S6A,B) nor did it sensitise WT cells to surplus extracellular cholesterol (Fig. S6C,D). Likewise, the loss of SOAT1 activity (+AVA) did not alter SQS levels (Fig. S6E) and AVA-treated cells grew normally in LPDS (Fig. S6F). These data point to the simultaneous loss of SQS and SOAT1 independently of cholesterol levels, such that their combined deficiencies produce a unique state of cholesterol homeostasis within EMC-deficient cells. The absence of change in mRNA levels (Figs S6G, S4M) indicates that attenuation of both SQS and SOAT1 occurs at least post-transcriptionally and likely post-translationally.

### SQS insertion by EMC enables sterol biosynthesis

**Fig. 6.**
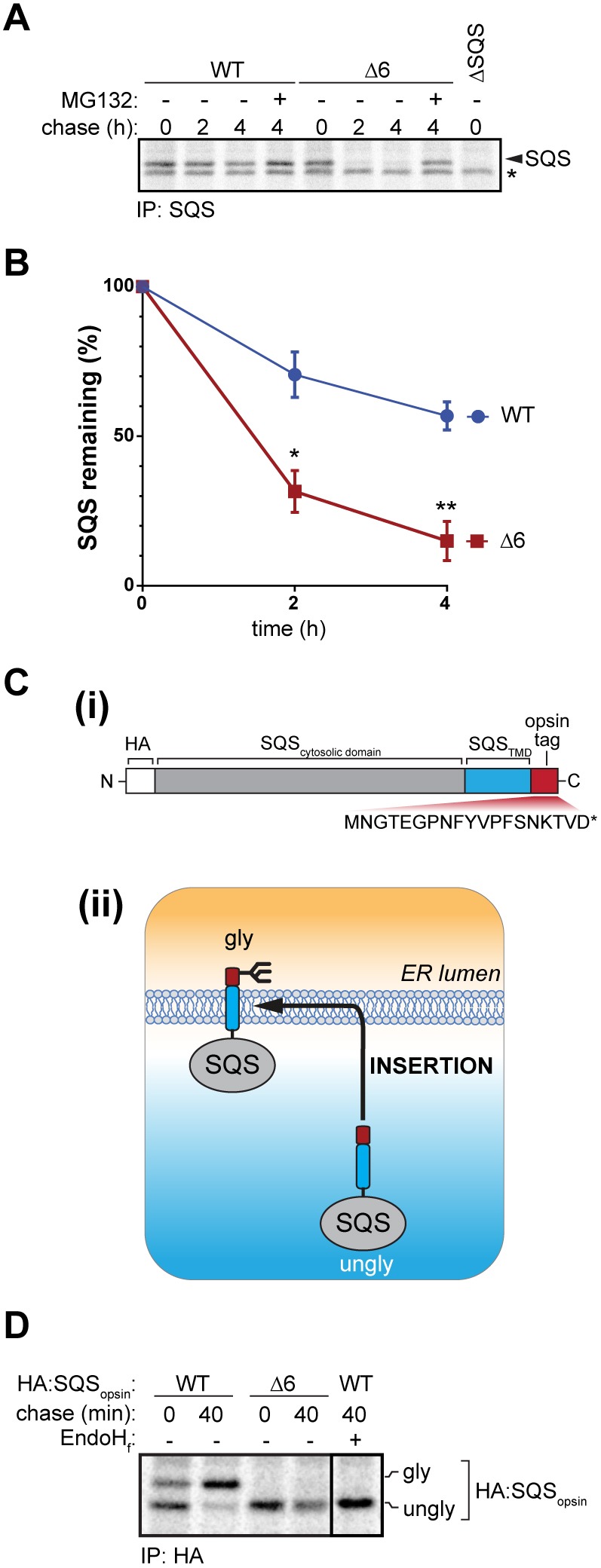
**The EMC is required for membrane insertion of SQS.** (A) ^35^S-Met/Cys pulse-chase assay of endogenous SQS in WT and Δ6 cells (0, 2, 4 h). Where indicated, cells were treated with MG132 (10 µg/ml, 4 h). Labelling of ΔSQS cells served as a negative control for immunoprecipitations (IPs). Nonspecific bands are indicated (*). Radiolabelled SQS was immunoprecipitated, separated by SDS-PAGE and bands quantified by phosphorimaging. (B) Quantification of SQS pulse-chase assays from three independent experiments performed as in A. Means±s.d. are shown. *P≤0.05, **P≤0.01 (Student's *t*-test). (C) (i) An epitope-tagged SQS variant C-terminally fused to an opsin tag (HA:SQS_opsin_) was used to monitor insertion efficiency into the ER membrane. Opsin primary sequence and the glycosylation site (red) are shown. (ii) Unglycosylated (ungly) HA:SQS_opsin_ is synthesised in the cytosplasm and glycosylated (gly) upon membrane insertion. (D) ^35^S-Met/Cys pulse-chase insertion assay of HA:SQS_opsin_ inducibly expressed in WT and Δ6 cells. Cells were collected 0 and 40 min after radiolabelling and HA:SQS_opsin_ was immunoprecipitated. Where indicated, eluates were treated with EndoH_f_ to confirm glycosylation. Core glycosylated (gly) and unglycosylated (ungly) HA:SQS_opsin_ are shown.

To determine the mechanism responsible for downregulating SQS in EMC-knockout cells, we monitored endogenous SQS synthesis and turnover by performing ^35^S-Met/Cys pulse-chase assays. SQS was degraded ∼3.5-fold faster in ΔEMC6 cells when compared to WT (*t*_1/2_=1.3 h versus 4.7 h, respectively, Fig. 6A,B), and the effect could be mitigated by addition of MG132 (Fig. 6A). Importantly, SQS was not stabilised in ΔEMC6 cells upon treatment with the VCP/p97 inhibitor NMS-873 (Fig. S6H), indicating that it was not degraded by the canonical ERAD pathways when the EMC was absent.

SQS is a tail-anchored (TA) membrane protein that must be inserted post-translationally into the ER. TA proteins failing to insert in the ER membrane are typically degraded (Borgese and Fasana, 2011). The EMC was recently shown to be necessary and sufficient for SQS TMD insertion into the ER membrane (Guna et al., 2018). We confirmed this dependency by using an SQS variant with a C-terminal opsin tag that is able to accept N-linked oligosaccharides (Brambillasca et al., 2005) (Fig. 6C) and repeated the pulse-chase studies. In WT cells, SQS was converted from a non-glycosylated precursor into a glycosylated product within 40 min. By contrast, very little glycosylated SQS was detected in ΔEMC6 cells (Fig. 6D). Importantly, glycosylation of an SQS variant containing the TMD from another TA protein, the well-studied Sec61β (Mariappan et al., 2010) (SQS-Sec61β^TMD^), was not impaired (Fig. S6I). These data show that the EMC is responsible for inserting the TMD from SQS and that oligosaccharyltransferase (OST) complex activity is not compromised by EMC deficiency. We asked whether bypassing EMC-dependent insertion of SQS could rescue the viability of ΔEMC cells when depleted of extracellular cholesterol. But although ΔEMC6 cells were able to insert SQS-Sec61β^TMD^ into the ER, this chimera could not restore viability following the depletion of extracellular cholesterol, perhaps indicating that the SQS TMD is essential for function or that additional EMC-dependent disruptions to sterol-related factors are involved downstream of SQS. These data demonstrate that the TMD of SQS directs its proper insertion by the EMC. Furthermore, insertion by the EMC is essential for SQS to become functional through appropriate ER membrane localisation that allows it to reach expression levels sufficient to provide robust sterol biosynthesis.

## DISCUSSION

### Critical roles for subunits in EMC assembly

Individual subunits of the EMC have been implicated in regulation of various proteins and processes, but how those activities are linked to the fully assembled, mature EMC oligomer had not been fully appreciated. In a systematic targeted knockdown screen for all 10 EMC subunits in multiple cell lines, we identified EMC6, EMC5 and EMC2 as essential for the intrinsic stability of other subunits within the EMC oligomer (Fig. 1). This observation implies first, that these ‘core subunits’ play a central role in EMC maturation. Secondly, disrupting individual core subunits confers loss of the entire EMC and any associated activity (Louie et al., 2012). Finally, roles attributed to most of the individual subunits, at least the essential ones, probably reflect activity of the fully assembled EMC. Robust phenotypes in model systems have been reported with the loss of individual EMC subunits (Richard et al., 2013; Satoh et al., 2015). Moreover, multiple siRNA and CRISPR/Cas9 loss-of-function screens for host factors supporting flavivirus infection/replication or cytotoxicity have identified more than one EMC subunit (Ma et al., 2015; Marceau et al., 2016; Savidis et al., 2016; Zhang et al., 2016). Among the strongest hits in these screens were EMC5 and EMC6, consistent with the essential roles in EMC maturation that we have defined.

The interdependency demonstrated by subunits in order to maintain EMC integrity complicates the deconvolution of their individual contributions to its activity. Notwithstanding, we did find that depletion of subunits outside the core (i.e. EMC8, EMC9 or EMC10) did not markedly affect SQS maturation (Fig. S5J,K), suggesting that only selected subunits within the EMC oligomer contribute directly to inserting (or chaperoning) client TMDs. EMC activity could be derived from the coordinated contributions of multiple subunits or could be mediated by a single subunit, such as EMC3, which recent bioinformatic evidence has identified as a member of the Oxa1/Alb3/YidC family of membrane protein biogenesis factors (Anghel et al., 2017; Borowska et al., 2015). It is worthwhile noting that EMC4 depletion did not destabilise other subunits but did preclude maturation of SQS (Fig. S5J,K) and other TMD-containing proteins (Shurtleff et al., 2018), and also suppressed flavivirus infection (Savidis et al., 2016), reflecting an important role in EMC functionality. By systematically defining the approximate architecture of the EMC and interdependency of its subunits, previous and future studies of this complex can be interpreted with greater precision. Ultimately, a more detailed structure–function analysis of the mammalian EMC will be required to differentiate the distinct roles and contributions of each subunit.

### SQS and SOAT1 biogenesis require the EMC

**Fig. 7.**
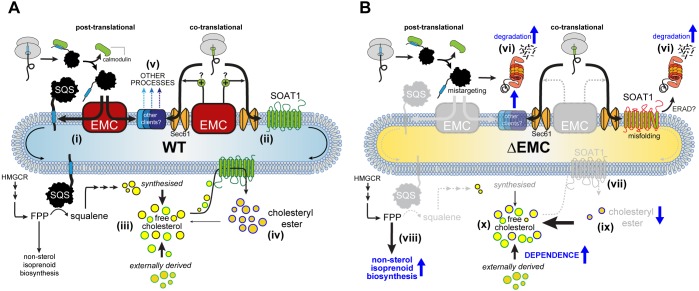
**Model of how EMC-mediated protein biogenesis influences cholesterol homeostasis.** (A) The EMC promotes the post-translational insertion of SQS into the ER membrane (i) and maturation of SOAT1 co-translationally (ii). The correct insertion and/or maturation of EMC-dependent proteins maintain cholesterol metabolic pathways by ensuring sufficient expression of enzymes essential for cholesterol biosynthesis and storage in the form of cholesteryl esters (iii, iv). These activities enable the biosynthetic and import pathways to maintain sufficient free cholesterol in cells. Additionally, the EMC is involved directly (or indirectly) in the biogenesis of other putative membrane-bound ‘clients’ with a variety of cellular roles (v). (B) Absence of the EMC precludes SQS insertion, resulting in its premature degradation via the proteasome (vi). Similarly, SOAT1 expression is also reduced co-/post-translationally due to defective biogenesis (vi, vii). The reduction of steady-state levels of both enzymes (depicted in grey) leads to increased utilisation of the mevalonate pathway intermediate FPP for non-sterol isoprenoid biosynthesis (viii), decreased cholesterol biosynthesis and reduced capability to store cholesterol as cholesteryl ester (ix), while at the same time maintaining sufficient free cholesterol to support viability (x). The consequential loss of other EMC clients may be manifest as non-lethal impairments detectable only when targeted or monitored directly.

Until recently, the molecular mechanism and the physiological role(s) for the ubiquitously expressed and evolutionarily conserved EMC had not been fully elucidated. However, a growing body of work has increasingly consolidated that it has a direct involvement in the biogenesis of both TA and polytopic membrane proteins in the ER. Here, we provide evidence that this EMC activity is a determinant of the homeostatic boundaries for cholesterol set within mammalian cells. EMC deficiency reduces cholesteryl ester stores but does not compromise the total free cholesterol in cells, nor does it trigger activation of SREBP2. However, the homeostatic challenges posed by growth in lipoprotein-depleted serum or exposure to high levels of extracellular cholesterol normally tolerated through robust sterol response mechanisms, become lethal without the EMC insertase activity. We traced the molecular basis of this striking phenotype in EMC-deficient cells to insufficient biogenesis and steady-state levels of at least two membrane-bound cholesterol homeostatic enzymes, SQS and SOAT1 (Fig. 7). Both SOAT1 and SQS were among proteins identified through quantitative proteomics and direct detection as strongly downregulated upon loss of the EMC. In contrast to the marked reduction observed for SQS (Fig. 5A,C–E), SOAT1 was not detected among the downregulated proteins by our proteomic analyses, perhaps due to an insufficient depth of coverage. Reductions in both SQS and SOAT1 protein levels were, however, reported in another proteomic analysis of EMC clients (Shurtleff et al., 2018), with SOAT1 also reduced in epithelial cells derived from EMC3-knockout mice (Tang et al., 2017).

SQS and SOAT1 differ significantly in their overall topology and mode of targeting to the ER membrane. SQS contains a single TMD at its C-terminus necessitating its insertion post-translationally into the ER membrane. SOAT1 contains nine (or more) TMDs (Guo et al., 2005) of varying hydrophobicity (Fig. S7A,B) and is inserted co-translationally by the Sec61 complex, potentially with assistance from the auxiliary translocon-associated protein (TRAP) complex (Nguyen et al., 2018). These are just two examples but it raises intriguing questions of how the EMC is able to equally promote the biogenesis of topologically diverse clients entering the ER through different pathways. In the case of SQS, the EMC is both necessary and sufficient for optimal insertion into the ER membrane, arising from its preference for TMDs of TA proteins with weak to moderate hydrophobicity (Guna et al., 2018). Without the EMC, SQS insertion into the ER membrane is no longer favoured, resulting in proteasome-dependent degradation, which is consistent with giving rise to the cholesterol auxotrophic phenotype.

For the polytopic SOAT1, the precise step at which the EMC is required during its biogenesis is less clear. Reduction of endogenous SOAT1 protein, but not mRNA, and the ability to rescue exogenous SOAT1 by proteasome inhibition (Fig. 4F) is consistent with a co-translational role for the EMC. Such a co-translational role for the EMC during biogenesis of polytopic multi-pass proteins with TMDs enriched with charged and aromatic residues has been recently described (Shurtleff et al., 2018). Moreover, during the preparation of this manuscript, EMC involvement in polytopic GPCR biogenesis was reported and revealed its requirement for the accurate insertion of the β-adrenergic receptor's first TMD into the ER membrane (Chitwood et al., 2018). Necessarily, both examples highlight cooperation with the Sec61 translocon during biogenesis of polytopic clients. Without the EMC, an unstable or suboptimal TMD encountered during membrane integration of the SOAT1 nascent chain could cause sufficient disruption to the folding programme and trigger degradation, potentially through ERAD (Shurtleff et al., 2018). Thus, we suspect that without the EMC, one (or more) SOAT1 TMDs may cause catastrophic folding failure during biogenesis, leading to premature degradation, loss of cholesterol esterification capacity and underlying cholesterol-induced cytotoxicity. The first crystal structure from the membrane-bound O-acyltransferase (MBOATs) superfamily, which includes SOAT1, has revealed a ring of 11 peripheral transmembrane helices in orientations that suggest synthesis and folding of this enzyme class may be complex (Ma et al., 2018). Further insight into the folding programme and structure of SOAT1 will be required to identify at which step the insertase activity of the EMC becomes critical.

### Setting the boundaries of cholesterol homeostasis

Through its role in facilitating SQS and SOAT1 biogenesis, our data indicate that the insertase activity of the EMC serves as an important determinant of the boundaries of cholesterol homeostasis. Independently losing either SQS or SOAT1 activity is sufficient to invoke well-defined compensatory homeostatic responses, and so it was surprising that transcriptional feedback mechanisms (via SREBP-2) were not strongly activated in EMC-deficient cells (Fig. 3A,B; Fig. S3) nor were the levels of the key cholesterol-related factors LDLR and HMGCR elevated (Fig. 3B,D). The level of SM was inversely correlated with the EMC, but that could be attributable to multi-layered post-translational regulation of SM stability via unsaturated fatty acids (Stevenson et al., 2014) and cholesterol (Gill et al., 2011). In order to maintain homeostasis with attenuated cholesterol biosynthesis and storage capabilities, EMC-deficient cells instead adapt, possibly by redistributing available cholesterol resources (Figs 3D, 4B, 7). This scenario may be uniquely created, at least in part, upon the coincidental loss of SQS and SOAT1. Without sufficient capacity to esterify and store cholesterol taken up (by LDLRs), we posit that it could become available to offset attenuated biosynthetic productivity rather than becoming cytotoxic. With homeostasis maintained, but at the cost of its cellular cholesterol reserves, the adapted state of EMC-deficient cells are precarious and at risk to cholesterol fluctuations that would otherwise be survivable. While the sensing and transcriptional response mechanisms for cholesterol change remain intact, their implementation is diminished because optimal expression of key responsive enzymes requires the EMC. Consequently, cell viability can only be maintained within a much narrower window of cholesterol availability.

It is worth noting that several other cholesterol-related proteins are also affected by EMC loss. Among them were ABCA3 (Tang et al., 2017) and TMEM97 [this study (Table S3) and Shurtleff et al., 2018], both of which are membrane proteins linked with intracellular cholesterol trafficking. ABCA3 is a cholesterol-binding protein/transporter present in a population of late endosomes linked with cholesterol transport/handling across the plasma membrane (van der Kant et al., 2013). TMEM97 is the recently identified σ2 receptor (Alon et al., 2017) that binds to the intracellular cholesterol trafficking protein NPC1 (Niemann-Pick disease, type C1), reducing its abundance and in turn cholesterol transfer to the ER (Ebrahimi-Fakhari et al., 2016). Increasing cholesterol transfer from endosomes to the ER might be how EMC-deficient cells offset biosynthetic attenuation and still maintain apparent cholesterol homeostasis. Clearly, our study and others find that multiple enzymes related to cholesterol processing all share the common requirement of the EMC to facilitate their expression.

### Pleiotropic effects arising from EMC loss

Our data suggest a strategy for how to trace complex phenotypes from EMC disruption (Ma et al., 2015; Richard et al., 2013; Satoh et al., 2015; Savidis et al., 2016; Zhang et al., 2016) to specific events at the ER, where the EMC resides and its activity is manifest. This will no doubt be a challenging task given the indirect disruptions to key regulatory or metabolic processes such as dysregulated cholesterol-dependent cellular pathways and to the cytosolic factors stabilised by EMC-dependent membrane proteins. Additionally, the increased FPP availability arising from insufficient SQS activity might also contribute to the phenotypes observed with EMC loss (Fig. S5F). For example, irreversibly modifying proteins with FPP (or GGPP) lipid anchors increases hydrophobicity, affects membrane association and modulates protein–protein interactions (Roskoski, 2003). Prenylated proteins play critical roles in cell signalling, growth, proliferation (i.e. Ras GTPases) and subcellular protein trafficking (i.e. Rab GTPases) (Wang and Casey, 2016). Elevated non-sterol isoprenoid production will also affect haem A, the polysaccharide carrier dolichol or the redox cofactor ubiquinone (coenzyme Q10) (Beytía and Porter, 1976; Haeuptle et al., 2011), but the consequences for these processes may only become apparent when monitored directly.

Deconvolving the contributions of individual EMC-dependent factors to complex phenotypes will require the uncoupling of function from protein biogenesis, a task that will be challenging until the entire range of EMC-dependent proteins can be identified. Our SILAC experiments, which identified SQS, provide an important step in this direction, but future studies will require greater depth to identify lower abundance proteins, such as SOAT1. It is worth mentioning that not all the downregulated proteins identified by SILAC from ΔEMC cells were integral membrane proteins. So while the EMC is directly responsible for inserting TMDs and maturation of a subset of integral membrane proteins, the secondary impact to cytosolic proteins they interact with, must also be recognised.

Our data support a model where the EMC exhibits intrinsic chaperone activity towards both TA and multi-pass client proteins to promote insertion of TMDs required for their correct maturation in the ER. Importantly, among the prominent EMC clients are proteins involved in cellular cholesterol biosynthesis and storage whose relative abundance set the boundaries of cholesterol homeostasis in mammalian cells. This study emphasises that both the primary impact on protein biogenesis and secondary modulation to cholesterol, post-translational modifications, the membrane environment and interacting proteins, together are likely to contribute to the reported phenotypes associated with EMC disruption. Further studies will be necessary to deconvolve their contributions within complex phenotypes.

## MATERIALS AND METHODS

### Plasmids and expression constructs

Full-length EMC5 and EMC6 cDNAs were cloned into the pcDNA5/FRT/TO vector (Thermo Fisher Scientific, Waltham, MA) using the BamHI and XhoI restriction sites for stable integration into U2OS Flp-In™ T-Rex™ (or Flp-In™ T-Rex™ 293) cells. An EMC6 variant containing an N-terminal FLAG-HA tag (DYKDDDDKLDGGYPYDVPDYA) was generated by PCR and subcloned into pcDNA5/FRT/TO. To generate TMD insertion reporters, an HA tag (YPYDVPDYA) was appended via a short linker (GGTG) to the N-terminus of the catalytic domain of SQS (amino acids 1–377), fused through a linker (GS) to the predicted TMD (underlined) and flanking residues of either SQS (SRSHYSPIYLSFVMLLAALSWQYLTTLSQVTED) or human Sec61β (SPGLKVGPVPVLVMSLLFIASVFMLHIWGKYT), followed by an opsin tag (MNGTEGPNFYVPFSNKTVD) to yield HA:SQS_opsin_ and HA:SQS-Sec61βTMD_opsin_, respectively. Each was subcloned into the pcDNA5/FRT/TO vector. The SOAT-1 reporter (EGFP-P2A-RFP-3xFLAG-SOAT1) was generated by appending a sequence encoding EGFP-P2A-RFP to 3xFLAG-SOAT1 in pcDNA5/FRT/TO, as has been reported previously (Chitwood et al., 2018).

### Chemicals and compounds

The following compounds were used: 25-hydroxycholesterol (25HC, Sigma-Aldrich, St. Louis, MO), DAPI (Sigma-Aldrich), aminooxybiotin (Biotium, Fremont, CA), aniline hydrochloride (Sigma-Aldrich), avasimibe (Sigma-Aldrich), cholesterol (Sigma-Aldrich), doxycycline (Sigma-Aldrich), filipin III (Santa Cruz Biotechnology, Dallas, TX), gel filtration markers kit (MWGF1000, Sigma-Aldrich), GGTI 298 trifluoroacetate salt hydrate (Sigma-Aldrich), Hygromycin B Gold (InvivoGen, San Diego, CA), IAA (iodoacetamide, Sigma-Aldrich), lipoprotein-depleted fetal bovine serum (LPDS, Kalen Biomedical, Germantown, MD), LMNG (lauryl maltose neopentyl glycol, Anatrace, Maumee, OH), methyl-β-cyclodextrin (MBCD, Sigma-Aldrich), MG132 (Merck Chemicals Ltd, Darmstadt, Germany), N-ethylmaleamide (NEM, Thermo Fisher Scientific), propidium iodide (Sigma-Aldrich), puromycin (Invivogen), SILAC Lys-8-Arg-10 kit (282986444, Silantes GmbH, Munich, Germany), sodium-meta-periodate (Sigma-Aldrich) and ZA (zaragozic acid A, Santa Cruz Biotechnology).

### Antibodies

The following antibodies were used in this study: ACC1 (Cell Signaling, Danvers, MA, rabbit pAb, #4190), 1:1000; AlexaFluor R488 anti-mouse IgG (H+L) (Invitrogen, donkey pAb, #A21202), 1:4000 [immunoblotting (IB)], 1:400 [immunofluorescence (IF)]; AlexaFluor R488 anti-rabbit IgG (H+L) (Invitrogen, goat pAb, #A11008), 1:4000 (IB), 1:400 (IF); anti-mouse IgG-HRP (Santa Cruz Biotechnology, goat, #sc-2005), 1:10,000; anti-rabbit IgG-HRP (Santa Cruz Biotechnology, goat, #sc-2030), 1:10,000; EMC1 (rabbit pAb, kind gift of Enilza Espreafico, Sao Paulo, Brazil), 1:1000; EMC2 (Proteintech, Rosemont, IL, rabbit pAb, #25443-1-AP), 1:2000; EMC3 (Abcam, Cambridge, UK, rabbit pAb, #ab175537), 1:500; EMC4 (Abcam, rabbit pAb, #ab123719), 1:1000; EMC5/MMGT1 (Abcam, rabbit pAb, #ab174366), 1:1000; EMC6 (Abcam, rabbit pAb, #ab84902), 1:1000; EMC7 (Abgent, San Diego, CA, rabbit pAb, #AP11145c), 1:500; EMC8 (Abcam, rabbit pAb, #ab180065), 1:500; EMC9 (Abgent, rabbit pAb, #AP5632b), 1:500; EMC10 (Abcam, rabbit pAb, #ab180148), 1:1000; HA (purified from hybridoma, mouse mAb, clone 12CA5), 1:1000; histone H3 (Abcam, rabbit pAb, ab1791), 1:5000; HMGCR (Atlas Antibodies, Bromma, Sweden, mouse mAb, clone CL0260), 1:1000; Hsp70 (Santa Cruz Biotechnology, goat pAb, clone K-20, #sc-1060), 1:1000; LDLR (R&D Systems, Minneapolis, MN, goat pAb, #AF2148), 1:1000; Rap1a (Santa Cruz Biotechnology, rabbit pAb, clone C-17, #sc-1482), 1:1000; SCD1 (Abcam, mouse mAb, clone CD.E10, #ab19862), 1:1000; SM (Proteintech, rabbit pAb, #12544-1-AP), 1:1000; SOAT1 (Santa Cruz Biotechnology, mouse mAb, clone A-11, #sc-136959), 1:1000; SQS (Abcam, rabbit mAb, #ab109723), 1:2000; SQS (Abcam, rabbit mAb, #ab195046), 1:200 (IF), 1:2000 (WB); SREBP2 (R&D Systems, goat pAb, #AF7119), 1:1000; tubulin (Sigma-Aldrich, mouse mAb, #T5168/b-5-1-2), 1:1000; and ubiquitin (Cell Signaling Technologies, rabbit pAb, #3933S), 1:1000.

### Cell culture

U2OS Flp-In™ T-Rex™ [kind gift of Mads Gyrd-Hansen, Oxford, UK, described in Fiil et al., (2013)], Flp-In™ T-Rex™ 293 (Invitrogen) and Ewing sarcoma A673 cells (kind gift of Udo Oppermann, Oxford, UK, originating from the ATCC) were all maintained in Dulbecco's modified Eagle's medium (DMEM, Gibco^®^ GlutaMAX^®^, Life Technologies) plus 10% v/v fetal calf serum (FCS, biosera, Kansas City, MO) and L-glutamine (2 mM, Life Technologies) in 5% CO_2_ at 37°C. Stable expression cell lines generated by Flp recombinase-mediated integration were continuously cultured in DMEM with 10% FCS and 100 µg/ml hygromycin B (Hygromycin B Gold, InvivoGen). All cell lines were routinely tested for mycoplasma contamination.

### Generation of stable Flp-In™ T-Rex™ cell lines

10^6^ cells per 6 cm tissue culture plate were co-transfected with the pcDNA™/5/FRT/TO plasmid containing the gene of interest (3 µg) and Flp recombinase (pOG44, 1 µg) using Lipofectamine™ 2000 (Thermo Fisher Scientific). The following day, cells were trypsinised and seeded in 10 cm tissue culture plates to achieve 20–40% confluency at 48 h after transfection. Cells stably integrating the gene of interest were selected by supplementing growth medium with hygromycin B for 7–10 days at the following concentrations; 250 µg/ml (U2OS Flp-In™ T-Rex™) or 100 µg/ml (Flp-In™ T-Rex™ 293).

### RNAi

To transiently silence expression of individual EMC subunits, the following ON-TARGETplus siRNA SmartPools (GE Healthcare/Dharmacon, Chicago, IL) (4×25 nmol) were used: EMC1 (LQ-014146-01-0002), EMC2 (LQ-010631-00-0002), EMC3 (LQ-010715-02-0002), EMC4 (LQ-021126-00-0002), EMC5/MMGT1 (LQ-018365-00-0002), EMC6 (LQ-014711-02-0002), EMC7 (LQ-021215-01-0002), EMC8 (LQ-032511-02-0002), EMC9 (LQ-017638-02-0002), EMC10 (LQ-018434-02-0002), non-targeting pool siRNA (D-001810-10-20). At 24 h after transfection, cells were expanded on 10 cm plates and incubated for an additional 48 h.

### CRISPR/Cas9-mediated genomic editing

CRISPR/Cas9-mediated genomic editing was performed in accordance with previously described protocols (Ran et al., 2013). Cells were transfected with a pSpCas9(BB)-2A-Puro (PX459) plasmid (Addgene 62988) containing an sgRNA targeting EMC2, EMC5, EMC6 or SQS (2–3 sgRNAs/gene, Table S1). Cells were grown for 48 h and subsequently treated with puromycin (PURO, Invivogen, 1 µg/ml) for 72 h to select transfected clones. Single-cell clones were isolated from PURO-resistant cells by limiting dilution. Disruption to gene expression was confirmed by western blotting of whole cell lysates (described below) and/or by genomic sequencing (Fig. S1G).

### Crystal Violet staining

To measure cell density via Crystal Violet incorporation, cells were fixed in cold methanol:acetone (1:1) (10 min, 4°C). After removing the fixative, cells were stained with a solution of 10% ethanol plus 0.1% (w/v) Crystal Violet (10 min), rinsed with ddH_2_O and air-dried. Representative micrographs were acquired by light microscopy and cell density per well was determined by imaging-based quantification of incorporated Crystal Violet dye by using an FLA-5100 laser scanner (Fujifilm Life Science, Singapore; 640 nm excitation wavelength, 25 µm resolution). The integrated pixel density of the resulting images was determined using the ImageJ software package.

### Cell viability

Cell viability was determined by collecting both detached and attached cells. Attached cells were collected by incubation with PBS plus 10 mM EDTA (10 min, 37°C). Cells were pelleted and resuspended in ice-cold PBS plus 3 mM EDTA. Prior to their analysis by flow cytometry, propidium iodide (PI) was added to cells (1 µg/ml final concentration). During the entire procedure, cells were kept on ice. PI incorporation was determined using a FACS Canto (BD Biosciences, Franklin Lakes, NJ). Data were analysed using the FlowJo (FlowJo, LLC, Ashland, OR) software package.

### RNA isolation, reverse transcription and qPCR

RNA was isolated from cells using the RNeasy Mini Kit (Qiagen, Venlo, Netherlands) according to the manufacturer's instructions. Reverse transcription was performed using the QuantiTect Reverse Transcription Kit (Qiagen). Transcript levels were measured in triplicate using the GoTaq^®^ qPCR Master Mix (Promega, Madison, WI) and an RT-PCR cycler (Rotor Gene 6000, Qiagen) with the following conditions: holding step (2 min, 95°C), melting step (72–95°C at 1°C/step), with pre-melt conditioning (90 s at the first step), 60°C annealing temperature, 40 cycles. Primer pairs used in this study are listed in Table S2. Relative mRNA levels were calculated using the ΔΔCT method. Transcript levels of actin were used for normalisation. Statistical significance was calculated with a Student's *t*-test.

### Sample preparation for RNA-Seq analysis

All cell lines used for RNA isolation are cultured under normal, nutrient-replete conditions (10% FCS in DMEM, 2 mM glutamine). Per sample, 5×10^6^ cells were seeded onto 10 cm tissue culture plates and cells were collected after 24 h. Then, 3 ml TRI Reagent was directly added to the plates, and lysates were snap-frozen and stored at −80°C. Total RNA isolation was performed from 1 ml cell suspension: after adding 200 µl chloroform, the suspension was centrifuged (17,000 ***g***, 10 min), followed by addition of 500 µl of chloroform to the upper phase, centrifugation at (17,000 ***g***, 5 min). Isopropanol (500 µl) was added to the upper layer and nucleotides were precipitated at −20°C (30 min), and the pellet collected by centrifugation (17,000 ***g***, 4°C, 5 min), rinsed with 80% ethanol and resuspended in 10.2 µl DEPC water. After DNase treatment (37°C, 30 min), 250 µl TRI Reagent was added and the chloroform extraction repeated as described above with adjusted volumes. The final RNA pellet was rinsed in 80% ethanol, dried and resuspended in 10 µl DEPC water.

### RNA-Seq analysis

Paired-end read alignment was performed using the HISAT2 (v2.1) (Kim et al., 2015) read aligner in strand-specific (RF) mode and the hg19 human reference genome. DESeq2 (Love et al., 2014) integrated in the SeqMonk package (v1.4 Simon Andrews and Laura Biggins, Babraham Bioinformatics) was used was used to determine differential gene expression.

### Flow cytometry

Cells cultured in the presence of DOX (100 ng/ml) were transfected with GFP-P2A-RFP-3xFLAG-SOAT1 (150 ng/well, 6-well plate) and incubated for 24 h before addition of MG132 (5 µg/ml) or DMSO (vehicle control) for 8 h. Flow cytometry was performed on a BD LSR Fortessa. 50,000 GFP-positive cells were collected and RFP:GFP ratios were determined using FlowJo (v.10). For quantification (Fig. 4G), mean RFP and GFP fluorescence intensities were determined in GFP-positive cells. RFP:GFP fluorescence ratios were calculated for each sample and normalised to the ratio in WT cells.

### Cholesterol starvation

Cells were seeded in 12-well tissue culture plates (10,000 cells/well) in DMEM plus 10% FCS. After 48 h, growth medium was replaced with DMEM containing 10% FCS and 4 mM MBCD (20 min, 37°C). Cells were rinsed three times with 1 ml PBS before addition of DMEM containing 5% FCS or 5% LPDS for 96 h and staining with Crystal Violet (see above). Alternatively, cells were seeded in a 96-well plate at 10,000 cells/well and after 24 h, MBCD was diluted in media collected from each well and added back (0–5 mM final concentration). After 16 h, cells were either stained with Crystal Violet or measured for the percentage of cell death by determining the level of PI incorporation using flow cytometry (see above).

### Preparation of Chol:MBCD complexes

Cholesterol (2.5 mM) was complexed with MBCD (25 mM) according to a protocol adapted from (Christian et al., 1997). Briefly, cholesterol powder (2.5 mM final) was added to an MBCD solution (25 mM). The resulting emulsion was vortexed and sonicated with a tip sonicator (1 min, 10 s intervals), followed by prolonged incubation (16 h, 37°C) under constant agitation. The solution was passed through a 0.45 µm PVDF syringe filter, aliquoted and stored at −20°C.

### Whole-cell lipidomic analysis

U2OS Flp-In™ T-Rex™ cells were seeded in 10 cm tissue culture plates (6×10^6^ cells/plate). After ≥18 h, cells were rinsed, harvested in ice-cold PBS and the resulting cell pellets stored at −80°C. Whole-cell extracts were prepared and lipids quantified in accordance with previous reports (To et al., 2017).

### SDS-PAGE and immunoblotting

Cells were mechanically collected in ice-cold PBS, centrifuged (1000 ***g***, 5 min, 4°C), and the resulting cell pellets resuspended in RIPA buffer [50 mM Tris-HCl pH 7.4, 150 mM NaCl, 0.1% (w/v) SDS, 1% (w/v) sodium deoxycholate, 1% (v/v) NP-40, 2 mM EDTA, 1× cOmplete™ Protease Inhibitor Cocktail (Roche), 1 mM PMSF]. Following incubation on ice (45 min), lysates were centrifuged to precipitate cellular debris (17,000 ***g***, 20 min, 4°C). Protein concentrations were determined by performing a Bradford assay; 6× Laemmli buffer and ∼20 mM DTT was added and lysates were denatured at 56°C (10 min). Protein samples were separated by SDS-PAGE and subsequently transferred to PVDF membranes (GE Healthcare) for immunoblotting. Membranes were blocked in PBST (PBS plus 0.1% Tween-20) containing 5% non-fat dry milk (60 min). Primary antibodies were incubated in PBST plus 2% BSA (1–3 h, 20°C or 16-24 h, 4°C). Membranes were incubated in PBST plus 5% non-fat dry milk containing either horseradish peroxidase (HRP)-conjugated or Alexa Fluor 488-conjugated secondary antibodies (1 h, 20°C) to allow detection of proteins by either ECL or fluorescence (described below).

### Densitometric analysis of immunoblots

Individual protein levels were quantified by densitometry from western blots using target-specific primary antibodies and secondary antibodies conjugated to Alexa Fluor 488. Images were acquired using the ChemiDoc system (Bio-Rad) and bands quantified with the ImageJ software package. Only signal intensities below the saturation threshold [as determined by the Image Lab 5.1 software (Bio-Rad)] were used for quantification. The area under the curve for the integrated pixel intensities of each band was measured for proteins of interest and internal tubulin (TUB) controls. Band intensities were normalised to TUB derived from the same sample.

### Affinity purification

Cells were collected as above and resuspended in lysis buffer (LB; 50 mM Tris-HCl pH 7.5, 150 mM NaCl, 5 mM EDTA, 2 mM NEM, 1× cOmplete™ Protease Inhibitor Cocktail) plus 1% Triton X-100 (v/v) or 1% LMNG (w/v) on ice (45 min). Detergent-soluble supernatants were isolated by centrifugation (17,000 ***g***, 4°C, 20 min) and pre-cleared with Sepharose CL-4B (100 μl, 50:50 slurry), (1–16 h, 4°C). Resulting lysates (0.3–1 mg) were used for affinity purification with HA-conjugated agarose beads (Roche) for 2 h (4°C). The resin was collected by centrifugation (1 min, 3000 ***g***, 4°C), washed (20× bead volume) once with high-salt buffer (50 mM Tris-HCl pH 7.5, 500 mM NaCl, 5 mM EDTA, 2 mM NEM) and twice with LB. Bead-bound proteins were resuspended in 2× Laemmli buffer plus 20 mM DTT, heated (10 min, 56°C) and separated by SDS-PAGE.

### SILAC-MS/MS

#### SILAC labelling and cell surface biotinylation

The PMP procedure was adapted from Weekes et al. (Weekes et al., 2012). Cells were maintained for ≥6 passages in media supplemented with isotopically labelled ‘light’ (K0/R0), ‘medium’ (K4/R6) or ‘heavy’ (K8/R10) amino acids. Cells were grown in 15 cm tissue culture plates, washed twice with ice-cold PBS (pH 6.7) and labelled with 1 mM sodium meta-periodate, 10 mM aniline and 100 mM aminooxy-biotin plus PBS (pH 6.7) under constant agitation in the dark (90, 4°C). The reaction was quenched by addition of 1 mM glycerol (30 min). The reaction mixture was removed and cells were washed with PBS (pH 7.4) plus 5% FCS followed by PBS (pH 7.4) containing 1 mM CaCl_2_ and 0.5 mM MgCl_2_.

#### Sample preparation for MS/MS

Cells were collected in ice-cold PBS using a cell strainer, centrifuged at 1500 ***g*** for 5 min and resuspended on ice in 300 µl of Triton X-100 lysis buffer [50 mM Tris-HCl pH 7.5, 150 mM NaCl, 1% (v/v) Triton X-100, 5 mM EDTA, 2 mM NEM, 1 mM PMSF, 1× Roche Protease Inhibitor Cocktail] for 45 min. Cell debris was removed by centrifugation (25 min, 17,000 ***g***, 4°C). Cell lysate from K0/R0, K4/R6 or K8R10 cells were equally mixed based on the Bradford assay results. 77.8 µl of 50% streptavidin/agarose slurry was added per mg of whole-cell lysate and incubated for 90 min at 4°C. Supernatant and bead-bound material were isolated by centrifugation and processed separately. Beads were washed using Micro Bio-Spin Chromatography Columns (Bio-Rad, Cat. No. #732-6204). All washing and modification steps were performed with 600 µl buffer and centrifugation steps were performed at 1000 ***g*** for 1 min at 4°C. Beads were consecutively washed twice with Triton X-100 lysis buffer and with PBS/0.5% (w/v) SDS. Disulfide bonds were reduced by incubation with PBS/0.5% (w/v) SDS and 100 mM DTT at room temperature for 20 min. Subsequently, beads were washed twice with UC buffer (100 mM Tris-HCl pH 8.5, 6 M urea) and treated with UC buffer with 50 mM iodoacetamide at room temperature for 20 min. The samples were successively washed twice with UC buffer, 5 M NaCl, 100 mM Na_2_CO_3_, PBS and H_2_O. Peptides were obtained after overnight incubation with trypsin at 37°C. The samples were acidified to 1% final concentration of trifluoroacetic acid (TFA). The peptides were collected in new tubes by centrifugation in SpinX spin column (Sigma Aldrich) at 1000 ***g*** for 1 min and were desalted using a C18 Sep-Pak cartridges (Waters, Milford, MA) according to the manufacturer's instructions. Briefly, each cartridge was conditioned with solution B (0.1% TFA in 65% CH_3_CN) and equilibrated with solution A (0.1% TFA in 98% H_2_O). Tryptic peptides were loaded and washed with solution A prior elution with solution B. The samples were dried in a vacuum concentrator and resuspended in 20 µl solution A.

#### Sample preparation of cytoplasmic fractions

The cytoplasmic fraction was obtained from the supernatant after affinity purification of biotinylated proteins. Proteins were enriched by chloroform/methanol precipitation and resuspended in 6 M urea, 0.1 M Tris-HCl pH 7.8. The solution was diluted 1:6 with H_2_O and proteins were digested using a 1:50 ratio (trypsin:protein). Tryptic peptides were purified as described above.

#### Peptide analysis by UHPLC-coupled mass spectrometry

Peptide analysis was performed by ultra-high performance liquid chromatography (Dionex Ultimate 3000 UHPLC, Thermo Fisher Scientific) coupled with tandem mass spectrometry (Q Exactive, Thermo Fisher Scientific) on a PepMap RSLC column (C18, 2 µm, 100 A, 75 µm×50 cm). Peptides were eluted on a gradient starting with 2% buffer B (0.1% TFA and 5% DMSO in CH_3_CN) at 3 min to reach 35% by 123 min at a flow rate of 250 nl/min. Data acquisition was performed at a resolution of 70,000 full-width half maximum at mass/charge of 400 with lockmass enabled (445.120025 *m*/*z*), top 15 precursor ion selection and dynamic exclusion of 27 s, and fragmentation was performed in higher-energy C-trap dissociation (HCD) mode with a normalised collision energy of 28.

#### Data processing

Raw MS files were processed by using MaxQuant v. 1.5.1.2 (Cox and Mann, 2008). The data was searched using the reviewed human database downloaded from Uniprot in April 2015. The search was performed using the following settings: two missed cleavages were allowed and the peptide ion tolerance was adjusted to 0.05 Da. Variable modifications included N-terminal acetylation, methionine oxidation and deamidation (NQ). Carbamidomethyl cysteine was defined as a fixed modification. The false discovery rate for peptides and proteins was set at 0.01 against a reversed decoy database. For protein quantification, unique and razor peptides were used with a minimum ratio count of 1. Re-quantify was enabled. Data generated by MaxQuant was further analysed using Perseus v. 1.5.0.31 (Tyanova et al., 2016). Reverse peptides, proteins only identified by site and potential contaminants were removed from the dataset. Normalised ratios for H:L and M:L were log2 transformed. Intensities were log_10_ transformed. Significance B values were calculated with Perseus. Proteins with a decrease in abundance of at least 30% were classified as downregulated. Proteins commonly downregulated by at least 30% in multiple cell lines were identified by using Perseus. Venn diagrams were generated with Adobe Illustrator CS6.

### ^35^S-methionine/cysteine pulse-chase

Pulse-chase assays of U2OS Flp-In™ T-Rex™ cells (WT/Δ6) were carried out according to a previously described protocol (Christianson et al., 2008). Briefly, cells were starved in methionine/cysteine-free DMEM (Lonza) plus 10% dialyzed FCS (10 min) and metabolically labelled by adding ^35^S-methionine/cysteine [Met/Cys, EXPRE^35^S^35^S Protein Labelling Mix (PerkinElmer); 150 μCi/10 plate] for 10 min. The medium was removed, then cells were rinsed with PBS and incubated in DMEM plus 10% FCS and methionine/cysteine (50 mM each) for the indicated times. Cells were collected and resuspended in LBplus 1% Triton X-100 and post-nuclear fractions pre-cleared overnight using unconjugated Sepharose beads. SQS was immunoprecipitated from the detergent-soluble fraction using 70 µl of 50% protein G resin (Roche) slurry and 2 µl of anti-SQS antibody (ab195046, see above). Immunoprecipitated material was resuspended in 2× Laemmli buffer plus 20 mM DTT and separated by SDS-PAGE, and the radiolabelled proteins visualised and quantified using a phosphoimager and the QuantityOne and Image Lab software packages (Bio-Rad). Relative protein levels were plotted as a function of time and protein half-lives [*t*_1/2_=τln(2)] were calculated using non-linear regression (exponential one-phase decay).

### Velocity sedimentation gradients

Whole-cell lysates were prepared in LB plus 1% (v/v) LMNG, and 0.5–1 mg post-nuclear detergent-soluble lysate were applied to continuous sucrose gradients (5–15%) generated using a GradientMaster 108 R (Biocomp; parameters: 5–15% sucrose, short, S1/1; *t*=2:50 min; angle=81.5; speed=15 rpm). Sucrose was dissolved in a physiological salt solution (150 mM NaCl, 50 mM Tris-HCl pH 7.4, 5 mM EDTA, 1 mM PMSF) plus 1% LMNG [36,000 rpm (OptimaTM L-100 XP, SW41 rotor; Beckman Coulter, Brea, CA), 16 h, 4°C]. A total of 13 fractions were collected and proteins concentrated by incubation with 0.61 M trichloracetic acid for 60 min on ice, washed twice with ice-cold acetone and resuspended in 200 µl 2× Laemmli buffer plus 20 mM DTT. Where necessary, samples were neutralised with 1 M Tris-HCl (pH 9). Samples were heated (10 min, 56°C) and separated by SDS-PAGE. Gel filtration standards (Gel Filtration Markers Kit, MWGF1000, Sigma Aldrich) were separated on similar gradients to estimate protein complex size and included: alcohol dehydrogenase (150 kDa), β-amylase (200 kDa), apoferritin (443 kDa) and thyroglobulin (663 kDa). Standards were processed as above and were detected by silver staining according to the manufacturer's instructions (Silver Stain for Mass Spectrometry, Pierce).

### Subcellular fractionation

Nuclear and cytoplasmic extracts were isolated according to a protocol adapted from Suzuki et al., (2010). Briefly, 4×10^6^ cells seeded in 10 cm plates were collected by trypsinisation. Cells were pelleted (5 min, 1500 ***g***, 4°C) and lysed by tituration with a P1000 pipette in cold NP40 buffer (PBS plus 0.1% NP-40) with 1 mM PMSF and 1× cOmplete™ Protease Inhibitor Cocktail (Roche) (450 µl/10 cm plate). Lysates were centrifuged (10 min, 17,000 ***g***, 4°C) and the resulting supernatants saved (cytoplasmic fraction). Pellets were resuspended in 1 ml cold NP40 buffer, centrifuged (17,000 ***g***, 10 min, 4°C) and resuspended in 150 µl NP-40 buffer (nuclear fraction). 30 µl 6×Laemmli buffer and ∼20 mM DTT were added to nuclear and cytoplasmic fractions prior to heating (5 min, 95°C). To fragment DNA, all samples were sonicated shortly and an additional 150 µl NP-40 buffer, 30 µl 6×Laemmli buffer and ∼20 mM DTT were added to the nuclear fractions. Equal volumes of cytoplasmic and nuclear fractions were used for SDS-PAGE.

### Indirect immunofluorescence confocal microscopy

Cells seeded on 12-mm glass coverslips (Nunc) were washed twice with PBS and fixed with 4% paraformaldehyde (PFA) for 20 min. PFA was removed by rinsing twice with PBS and cells were permeabilised with 0.1% (v/v) Triton X-100 in PBS (5 min), washed twice with PBS, and incubated in blocking buffer [0.2% (w/v) fish skin gelatin (FSG) plus PBS, 60 min]. Primary antibody [anti-SQS rabbit mAb, ab195046 (Abcam), 1:200] in blocking buffer was added (60 min), the coverslips were rinsed twice with PBS and incubated in the dark (60 min) with Alexa Fluor 488-conjugated secondary antibody (1:400 in blocking buffer). DNA was stained using 5 µg/ml DAPI before mounting in Fluoromount G (Southern Biotech, Birmingham, AL). For Filipin III staining, after fixation with 4% PFA, cells were rinsed three times with PBS before quenching residual PFA with 1.5 mg/ml glycine plus PBS (10 min). Cells were incubated with Filipin III (Santa Cruz Biotechnology, 25 µg/ml in PBS, 30 min) and rinsed three times with PBS. Coverslips were sealed with transparent nail polish. Antibody-bound proteins were visualised at an excitation wavelength of 488 nm and DAPI and Filipin III were imaged at 405 nm using an LSM 710 (Zeiss, Oberkochen, Germany) confocal microscope. Images were processed using ImageJ and Adobe Illustrator software.

### Statistical analysis

Statistical significance was calculated using Student's *t*-test (Holm-Sidak method for multiple comparisons) and defined as *P*≤0.05.

### Bioinformatic analysis

Primary amino acid sequences for human EMC1-10 and human SQS were obtained from UniProt (http://www.uniprot.org/), with common motifs annotated using Pfam (http://pfam.xfam.org/) (Finn et al., 2016), TMDs predicted by TOPCONS (http://topcons.net/) (Tsirigos et al., 2015) and N-linked glycosylation sites predicted by NetNGlyc 1.0 (http://www.cbs.dtu.dk/services/NetNGlyc/).

## Supplementary Material

Supplementary information
