# Supplementary material for: The ER membrane protein complex promotes biogenesis of sterol-related enzymes maintaining cholesterol homeostasis
Source: J Cell Sci. 2019 Jan 16;132(2):jcs223453. doi: 10.1242/jcs.223453 (PMC6362398; doi:10.1242/jcs.223453)
Supplement: Supplementary information [file joces-132-223453-s1.pdf]

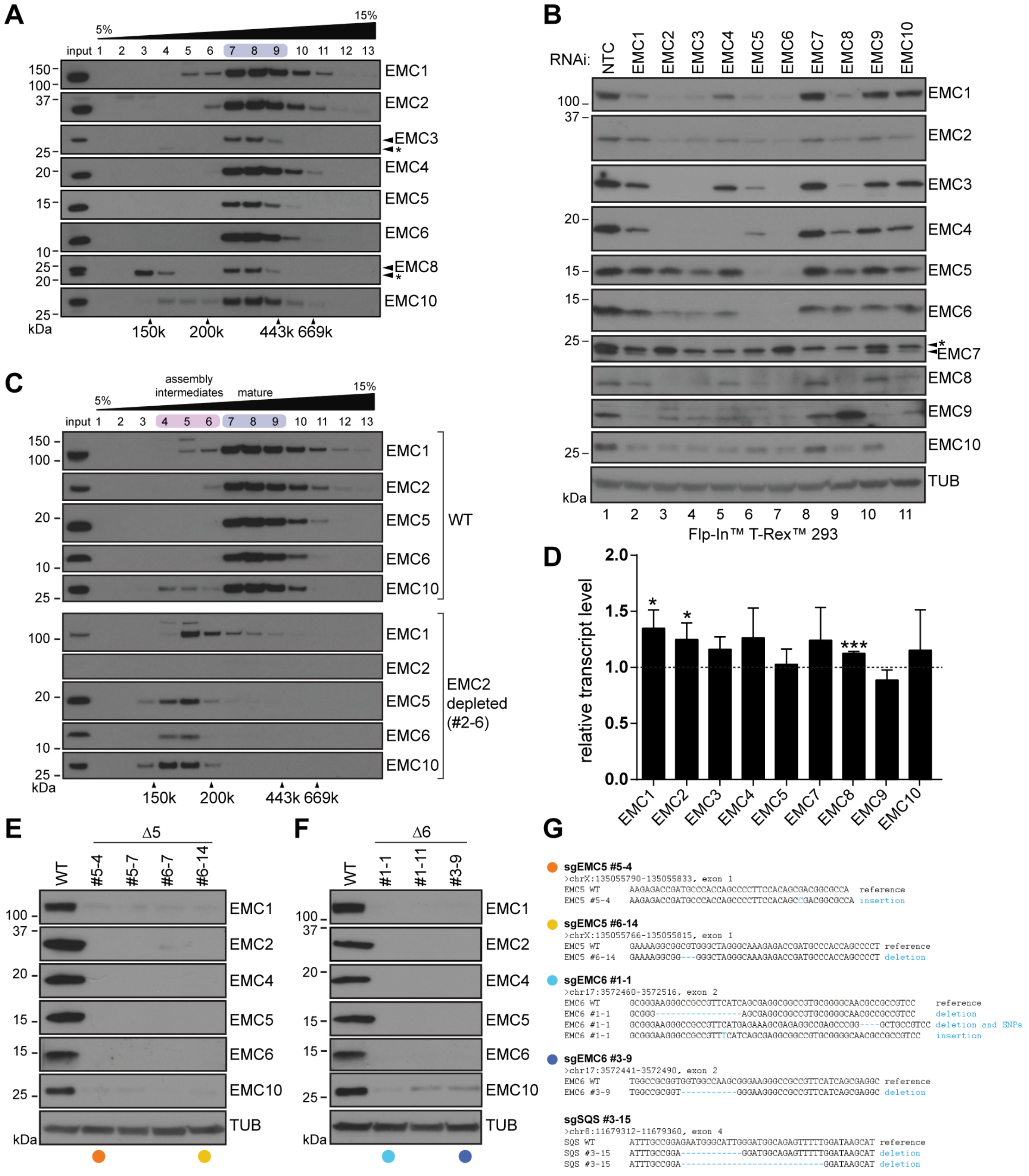

Figure S1. Contribution of individual subunits to EMC biogenesis.

(**A**) Lysates of U2OS Flp-In^{TM} TRex^{TM} cells solubilized in LMNG were separated by velocity sedimentation on a 5 – 15% sucrose gradient and collected as 13 fractions, revealing a single peak between 300 - 400 kDa (fractions 7-9, blue). Non-specific bands are denoted (*).

(**B**) Flp-In^{TM} TRex^{TM} 293 cells transfected with siRNAs targeting EMC1 – EMC10 or a non-targeting control (NTC) were collected after 72 h. WCL was separated by SDS-PAGE and resulting western blots probed with the indicated antibodies to EMC subunits.

Journal of Cell Science • Supplementary information

(**C**) Comparison of EMC sedimentation as in (A) from LMNG lysates of WT and EMC2-depleted cells. The mature EMC (fractions 7-9, blue) and putative assembly intermediates (fractions 4-6, red) are indicated and reflected by changes to sedimentation of EMC1/5/6/10.

(**D**) Transcript levels of non-targeted EMC subunits determined by qRT-PCR from U2OS Flp-In^{TM} TRex^{TM} cells depleted of EMC6 relative to non-targeting control (NTC)-treated cells as described in Figure 1B. Means ± S.D. ($n = 3$) are shown and significance determined by Students t-test: *$p \leq 0.05$, ***$p \leq 0.001$.

(E – G) Knockout of EMC5 and EMC6 by CRISPR/Cas9.

Western blots of WCL from selected U2OS Flp-In^{TM} TRex^{TM} clones knocked out for EMC5 (**E**, Δ5) or EMC6 (**F**, Δ6) by CRISPR/Cas9 using different sgRNAs and probed with antibodies to the indicated EMC subunits and tubulin (TUB). Colored circles indicate clone identity throughout the study. (**G**) Genomic sequencing of individual Δ5, Δ6 and ΔSQS clones used in this study.

Journal of Cell Science • Supplementary information

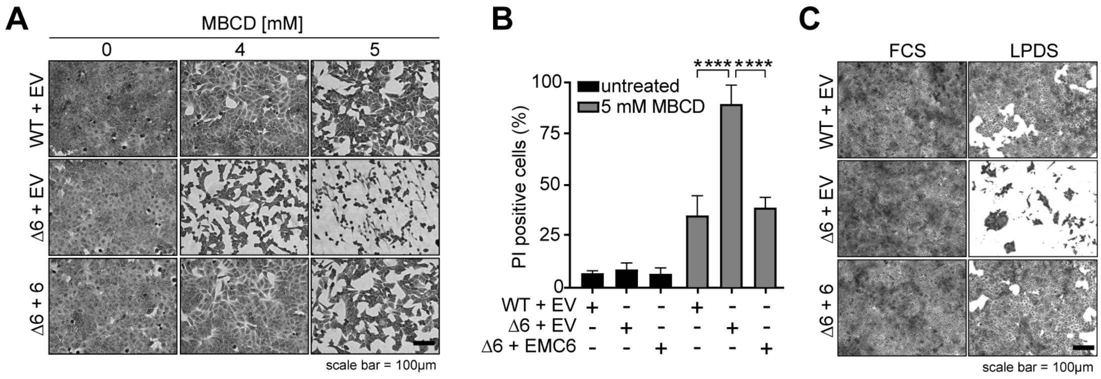

Figure S2. Cholesterol depletion induces cell death in EMC-deficient cells.

(**A**) WT and ΔEMC6 (Δ6) cells reconstituted with an empty vector control (WT + EV, Δ6 + EV) or EMC6 (Δ6 + 6) were treated with MBCD (4, 5 mM, 16 h) and stained with crystal violet. Scale bar = 100 μm.

(**B**) Quantification of propidium iodide (PI) incorporation into untreated and MBCD-exposed cells (5 mM, 16 h) as measured by flow cytometry. Means ± S.D. of PI positive cells are shown (n = 5) and significance determined by Students t-test: ****p ≤ 0.0001.

(**C**) Cells depleted of cholesterol by MBCD (4 mM, 20 min) were switched to FCS (5%) or LPDS (5%) containing growth media (6 d) and visualized by staining with crystal violet. Scale bar = 100 μm.

Journal of Cell Science • Supplementary information

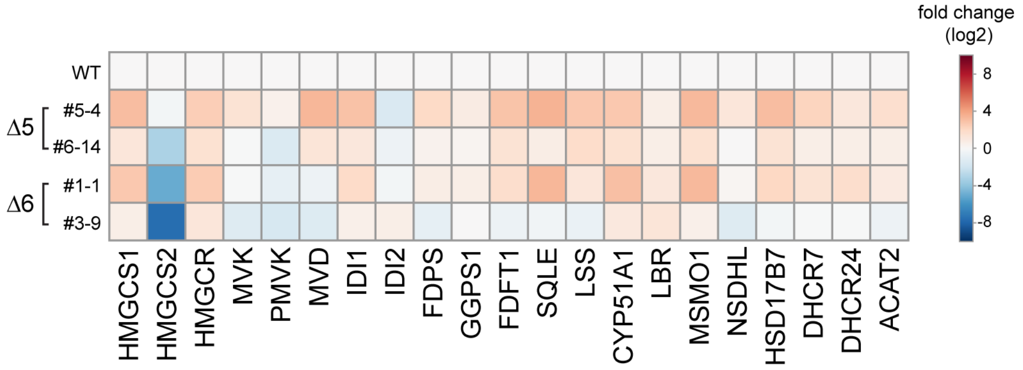

Figure S3. RNA-Seq analysis of genes involved in cholesterol biosynthesis.

Heat map of log2 transformed, normalised transcript levels of genes coding for proteins involved in cholesterol biosynthesis. U2OS WT, ΔEMC5 (Δ5, clones #5-4 and #6-14) and ΔEMC6 (Δ6, clones #1-1 and #3-9) cells were collected at steady-state. Data from three independent experiments (n = 3) are shown. HMGCS1/2, 3-Hydroxy-3-Methylglutaryl-CoA Synthase 1/2; HMGCR, 3-Hydroxy-3-Methylglutaryl-CoA Reductase; MVK, Mevalonate Kinase; PMVK, Phosphomevalonate Kinase; MVD, Mevalonate Diphosphate Decarboxylase; IDI1/2, Isopentenyl-Diphosphate Delta Isomerase 1/2; FDPS, Farnesyl Diphosphate Synthase; GGPS1, Geranylgeranyl Diphosphate Synthase 1; FDFT1, Farnesyl-Diphosphate Farnesyltransferase 1; SQLE, Squalene Epoxidase; LSS, Lanosterol Synthase; CYP51A1, Cytochrome P450 Family 51 Subfamily A Member 1; LBR, Lamin B Receptor; MSMO1, Methylsterol Monooxygenase 1; NSDHL, NAD(P) Dependent Steroid Dehydrogenase-Like; HSD17B7, Hydroxysteroid 17-Beta Dehydrogenase 7; DHCR7, 7-Dehydrocholesterol Reductase; DHCR24, 24-Dehydrocholesterol Reductase; ACAT2, Acetyl-CoA Acetyltransferase 2.

Journal of Cell Science • Supplementary information

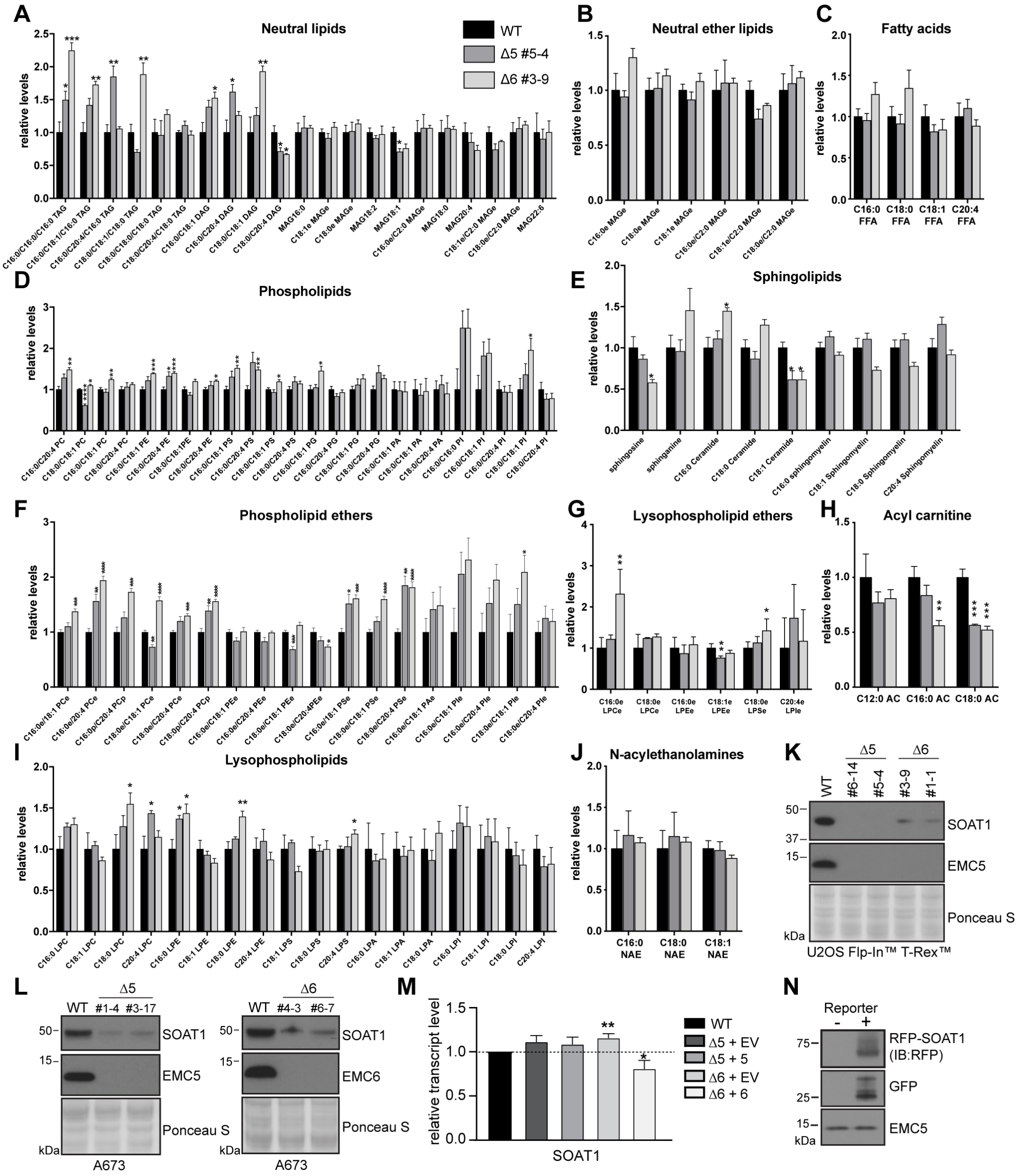

Figure S4. Relative abundance of major lipid species in EMC-deficient cells.

Lipid abundance in WT (black), ΔEMC5 (Δ5 #5-4) (dark grey) and ΔEMC6 (Δ6 #3-9) cells (light grey) was measured by LC-MS/MS. Means ± S.E.M. (n = 5) and significance (Students t-test: *p ≤ 0.05, **p ≤ 0.01, *** p ≤ 0.001, ****p ≤ 0.0001) are shown for: (**A**) neutral lipids, (**B**) neutral ether lipids, (**C**) fatty acids, (**D**) phospholipids, (**E**) sphingolipids, (**F**) phospholipid ethers, (**G**) lysophospholipid ethers, (**H**) acyl carnitine, (**I**) lysophospholipids, and (**J**) N-acylethanolamines.

(K - L) Reduced SOAT1 expression in EMC-deficient cell lines.

Western blots probed for endogenous SOAT1, EMC5 and EMC6 from EMC5 (Δ5) and EMC6 knockout (Δ6) clones generated from U2OS Flp-In^{TM} TRex^{TM} (**K**) or A673 cells (**L**).

(**M**) Relative SOAT1 mRNA levels in WT, EMC5/6 knockout cell lines reconstituted with an empty vector control (Δ5 + EV, Δ6 + EV) or EMC5/6 (Δ5 + 5, Δ6 + 6). Mean ± S.D. (n = 3) and significance are shown, Students t-test: *p ≤ 0.05, **p ≤ 0.01.

(**N**) Expression of GFP-P2A-RFP-3xFLAG-SOAT1 reporter in U2OS Flp-In^{TM} TRex + EV cells. Cells were treated as described in Figure 4F and whole-cell lysates separated by SDS-PAGE.

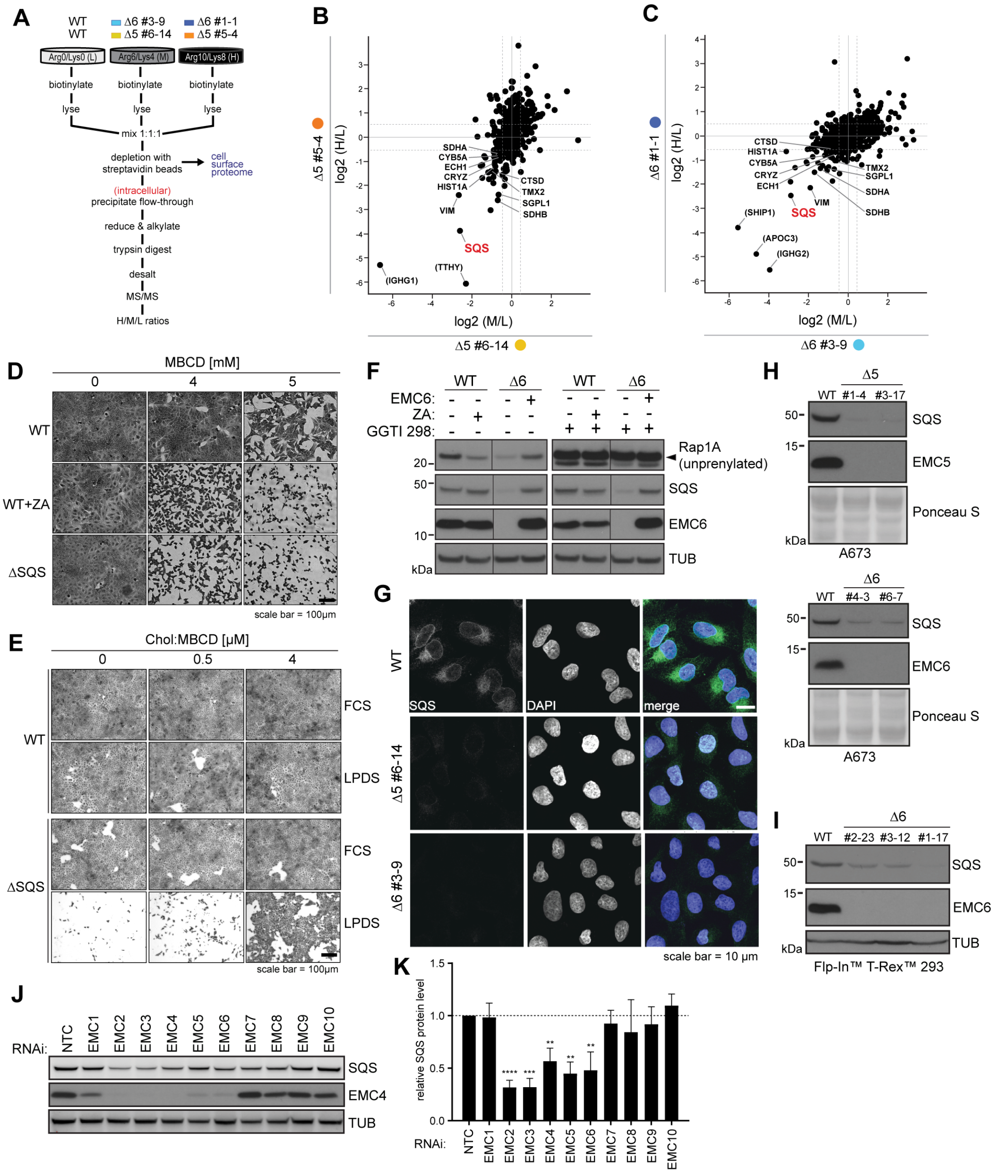

Figure S5. SQS biogenesis is EMC-dependent.

(A - C) Changes to intracellular protein levels accompanying EMC loss.

(**A**) Workflow for triple-label SILAC-MS/MS. Two individual U2OS Flp-In™ TRex™ cell lines deficient in either EMC5 (Δ5 #5-4, Δ5 #6-14) or EMC6 (Δ6 #1-1, Δ6 #3-9) were labeled with medium (R6/K4, M) or heavy (R10/K8, H) amino acids. Parental wild-type (WT) cells remained unlabeled (R0/K0, L). Intracellular fractions were enriched following affinity depletion of the cell surface sialoglycoproteome by aminooxy-biotin modification and streptavidin agarose. Signature peptides were identified by MS/MS and their relative abundance was determined by calculating M/L and H/L ratios. Scatter plots of all identified proteins in EMC5 knockout cells (Δ5 #5-4, Δ5 #6-14) (**B**) and EMC6 knockout cells (Δ6 #1-1, Δ6 #3-9) (**C**).

Journal of Cell Science • Supplementary information

Normalized H/L and M/L ratios were converted to a log2 scale and plotted against one another. Dashed lines demarcate the log2 = 0.5 threshold (corresponding to ≥30% change). Identified proteins are represented as single points, proteins decreased by ≥30% are annotated with gene names, and SQS is highlighted in red. Proteins not consistently reduced by ≥30% in all cell lines are shown in brackets.

(D – F) Cholesterol auxotrophy results from loss of SQS activity.

(**D**) Acute depletion of cholesterol from WT or ΔSQS cells by treating with MBCD (4, 5 mM, 16 h) and staining with crystal violet. Where indicated, WT cells were treated with zaragozic acid (ZA, 100 µM, 16 h) along with MBCD. Scale bar = 100 µm.

(**E**) Growth of ΔSQS cells in media containing 5% FCS or 5% LPDS ± Chol:MBCD (0, 0.5, 4 µM, 96 h) followed by staining with crystal violet. Scale bar = 100 µm.

(**F**) Western blots of WCL from WT, Δ6 ± EMC6 cells induced with DOX (1 ng/ml, 24 h), probed for unprenylated Rap1a. Where indicated, cells were treated with GGTI 298 (25 µM, 4.5 h) or zaragozic acid A (ZA, 10 µM, 4 h). SQS and EMC6 are shown for comparison. TUB serves as a loading control.

(G - K) SQS expression is compromised in EMC-deficient cells.

(**G**) Immunofluorescence images of endogenous SQS in WT and EMC-deficient cells collected by confocal microscopy. Scale bar = 10 µm.

(**H**) Western blot analysis of A673 single-cell EMC5 (left) or EMC6 knockout clones (right) generated by CRISPR/Cas9 and different sgRNAs. EMC subunits and SQS are shown.

(**I**) Western blot analysis of Flp-In^{TM} TRex^{TM} 293 WT and single-cell clones for CRISPR/Cas9-mediated knockout of EMC6. EMC6 and SQS are shown.

(**J**) siRNA-mediated knockdown of EMC subunits (72 h) in U2OS Flp-In^{TM} TRex^{TM} cells with resulting western blots probed for SQS, EMC4 and tubulin (TUB).

(**K**) Quantification of SQS from (J) by densitometry using Alexa 488 quantification and normalized to NTC (dashed line) are shown. Means ± S.D. (n = 3) are depicted and significance was determined by Students t-test: **p ≤ 0.01, ***p ≤ 0.001, ****p ≤ 0.0001.

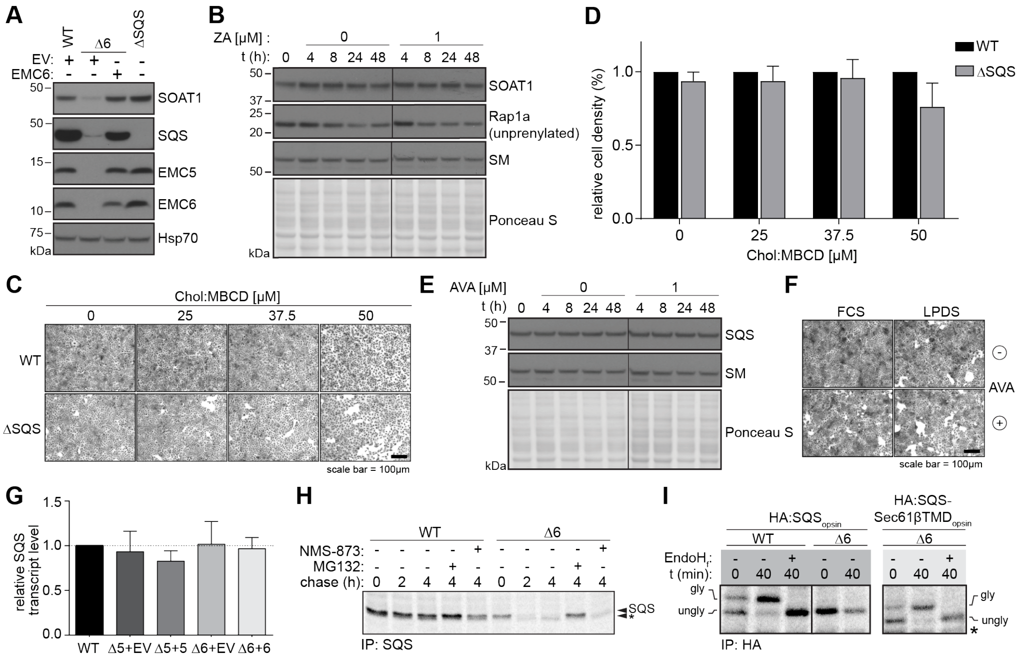

Figure S6. Loss of SQS and SOAT1 expression are not interdependent.

(**A**) SOAT1 western blot of lysates from WT, Δ6+EMC6 or EV and ΔSQS cells. Reconstitution was induced with DOX (10 ng/ml, 24 h). Hsp70 serves as a loading control.

(**B**) WT cells treated with zaragozic acid (ZA, 1 μM) for the indicated times with western blots of WCLs probed with antibodies to SOAT1, Rap1a (unprenylated) and SM.

(**C**) WT and ΔSQS cells were supplemented with Chol:MBCD (25, 37.5, 50 μM, 20 h) and stained with crystal violet. Scale bar = 100 μm.

(**D**) Quantification of three independent experiments performed as in (C). Means ± S.D. are shown.

(**E**) WT cells treated with avasimibe (AVA, 1 μM) for the indicated times with western blots of WCLs probed with antibodies to SQS and SM.

(**F**) WT cells grown in 5% FCS or 5% LPDS were treated with AVA (5 μM, 96 h) and visualized by crystal violet staining. Scale bar = 100 μm.

(**G**) Relative SQS transcript levels in Δ5 and Δ6 cells reconstituted with the missing subunit (Δ5 + 5, Δ6 + 6) or an empty vector control (Δ5 + EV, Δ6 + EV). Expression of EMC5/6 was induced with DOX (1 ng/ml, 24 h). mRNA levels were determined by qPCR and normalized to WT control (dashed line). Means and S.D. are shown (n = 3).

(H - I) Insertion of the SQS tail anchor is EMC-dependent.

Journal of Cell Science • Supplementary information

(**H**) ^{35}S-Met/Cys radiolabeled cells (WT, Δ6) were chased (2, 4 h) ± MG132 (10 µg/ml, 4 h) or NMS-873 (10 µM, 4 h). A representative IP of endogenous SQS is shown. Unspecific bands (*) are indicated.

(**I**) Pulse-chase assay performed as in Figure 6D with cells expressing either HA:SQS$_{opsin}$ or an HA:SQS$_{opsin}$ whose TMD was replaced with the TMD of Sec61β (HA:SQS-Sec61βTMD$_{opsin}$).

Journal of Cell Science • Supplementary information

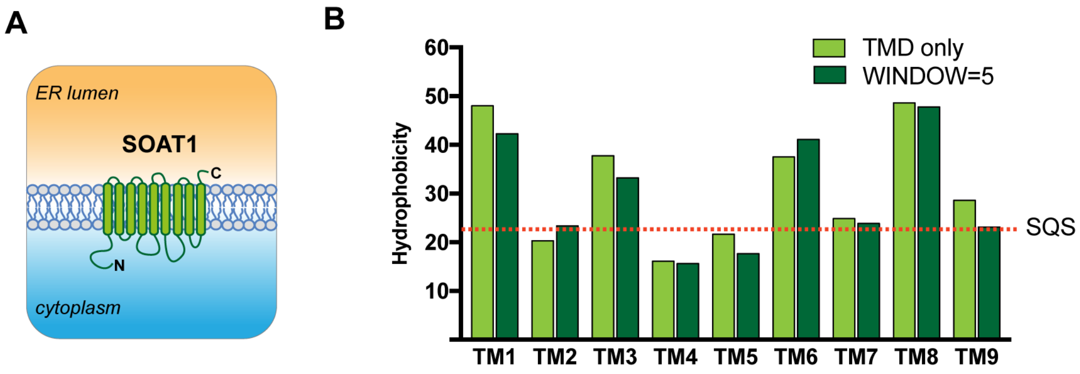

Figure S7. SOAT1 topology and in silico prediction of TMD hydrophobicity.

(**A**) Schematic representation of SOAT1 topology as reported previously (Guo *et al.*, 2007).

(**B**) In silico prediction of SOAT1 TMD hydrophobicity using the Kyte & Doolittle (K-D) method and calculated for the TMD only (light green) or using a sliding window (WINDOW = 5 aa, dark green) by ProtScale (https://web.expasy.org/protscale/). The calculated K-D value of the SQS TMD is indicated (dashed line). SOAT1 contains several TMDs whose K-D vales are lower (TM4, 5) or comparable (TM2, 7, 9) to that of the SQS TMD.

Table S1. gRNA sequences			
**gRNA**	**target gene**	**sequence**	**used to generate**
NV EMC2 2 (854)	EMC2	CACAGAGTCAAGCGATTAACAGG	U2OS Flp-In^{TM} TRex^{TM}: Δ2 #2-6
JC hMMGT 1 (410)	EMC5	GCATCATGGCGCCGTCGCTGTGG	U2OS Flp-In^{TM} TRex^{TM}: Δ5 #5-9<br>A673: Δ5 #1-4
JC hMMGT 2 (411)	EMC5	CGCAGCGGAAAAGGCGGCGTGGG	U2OS Flp-In^{TM} TRex^{TM}: Δ5 #6-14<br>A673: Δ5 #2-4
JC hMMGT 3 (412)	EMC5	CACTGCCAATAGATGTAAGTTGG	A673: Δ5 #3-17
JC hEMC6 1 (401B)	EMC6	GCCGCCTCGCTGATGAACGGCGG	U2OS Flp-In^{TM} TRex^{TM}: Δ6 #1-1<br>A673: Δ6 #4-3<br>Flp-In^{TM} TRex^{TM} 293: Δ6 #1-17
JC hEMC6 2 (402)	EMC6	CCGAGGTCCGGCAATAATCCAGG	Flp-In^{TM} TRex^{TM} 293: Δ6 #2-23
JC hEMC6 3 (403)	EMC6	GAACGGCGGCCCTTCCCGCTTGG	U2OS Flp-In^{TM} TRex^{TM}: Δ6 #3-9<br>A673: Δ6 #6-7, Δ6 #6-20<br>Flp-In^{TM} TRex^{TM} 293: Δ6 #3-12
NV FDFT1 1 (855)	SQS	GCCGACATTTGCCGGAGAATGGG	U2OS Flp-In^{TM} TRex^{TM}: ΔSQS #3-15

Journal of Cell Science • Supplementary information

Table S2. Primers used for qPCR

primer	sequence (5'-3')
Actin forward	GAGGCACTCTTCCAGCCTT
Actin reverse	AAGGTAGTTTCGTGGATGCC
EMC1 forward	GGAATGTCTTGCGACAGCTA
EMC1 reverse	AGATCCTTTCGAAGCCGATA
EMC2 forward	TCCAACTAACACTGCTGCAA
EMC2 reverse	AAGTTCATGCCAGGCTTCTT
EMC3 forward	GCCAAGATAATGCCGCTGAC
EMC3 reverse	TCGAAGTGGAGGTCTTTGGC
EMC4 forward	TTCAGCCACTTTCAAGATGTTAG
EMC4 reverse	GTCCTCCACCACTGAACTCC
EMC5 forward	CTGCGCAGCATCGTTCTTAT
EMC5 reverse	GCCGGAAAAGTACTCGACCA
EMC6 forward	GCCGCCGTCCTGGATTATT
EMC6 reverse	GGAGGCGAGCAGGTAGAAGA
EMC7 forward	CTGACATGAGACGGGAAATG
EMC7 reverse	GCTGCTAGATTTGCCAGATG
EMC8 forward	CTGCGCTCATCATGGTAGAC
EMC8 reverse	CCATCTGTTCTCATGGTGCT
EMC9 forward	TCAACCAGGTGGATGTGTGG
EMC9 reverse	GACCTTGGTTCTCCAGGACG
EMC10 forward	TGAGATCGATGACAGTGCCAA
EMC10 reverse	GCAGGGACAAAGGAGGAGAC
HMGCR forward	GGACCCCTTTGCTTAGATGAAA
HMGCR reverse	CCACCAAGACCTATTGCTCTG
SM forward	CTATGGCAGAGCCCAATGCAAAGT
SM reverse	ACAACAGTCAGTGGAGCATGGAGT
SQS forward	GGCAAGCGGAAGGTGATG
SQS reverse	CTGGTCTGATTGAGATACTTGTAGCAA
SOAT1 forward	GATGAAGGAAGGCTGGTGC
SOAT1 reverse	GGAAGCTGGTGGCAGTGTAT

Journal of Cell Science • Supplementary information

Table S3. Proteins reduced in EMC5 KO or EMC6 KO only

Reduced in EMC5 KOs only

Majority Protein IDs	Protein IDs	Also detectable in EMC6 KOs vs WT?	Ratio M_EMC5KO#6-14 / L_WT normalized	Ratio H_EMC5KO#5-4 / L_WT normalized
BAP31	sp|P51572|BAP31_HUMAN	Y	0.45265	0.30205
BGAL	sp|P16278|BGAL_HUMAN	N	0.6871	0.60476
CERU	sp|P00450|CERU_HUMAN	N	0.53153	0.2684
DBLOH	sp|Q9NR28|DBLOH_HUMAN	Y	0.34031	0.44166
DHRS2	sp|Q13268|DHRS2_HUMAN	Y	0.67075	0.64772
DHX9	sp|Q08211|DHX9_HUMAN	Y	0.67481	0.57474
EMC2	sp|Q15006|EMC2_HUMAN	N	0.48552	0.12459
GGH	sp|Q92820|GGH_HUMAN	Y	0.64931	0.6523
GNS	sp|P15586|GNS_HUMAN	N	0.52858	0.31681
HM13	sp|Q8TCT9|HM13_HUMAN	Y	0.56249	0.69006
HNRDL	sp|O14979|HNRDL_HUMAN	Y	0.539	0.68775
IF4G1	sp|Q04637|IF4G1_HUMAN	Y	0.35945	0.4535
K1C18	sp|P05783|K1C18_HUMAN	Y	0.67854	0.50497
NNRE	sp|Q8NCW5|NNRE_HUMAN	N	0.54377	0.57032
NSF	sp|P46459|NSF_HUMAN	Y	0.68141	0.63786
PTN1	sp|P18031|PTN1_HUMAN	Y	0.40108	0.55274
RISC	sp|Q9HB40|RISC_HUMAN	N	0.56403	0.49308
RL35	sp|P42766|RL35_HUMAN	Y	0.38315	0.58226
RM09	sp|Q9BYD2|RM09_HUMAN	Y	0.56882	0.55345
TTHY	sp|Q5U7I5|TTHY_PANTR	N	0.2013	0.015316

Reduced in EMC6 KOs only

Majority Protein IDs	Protein IDs	Also detectable in EMC5 KOs vs WT?	Ratio M_EMC6KO#3-9 / L_WT normalized	Ratio H_EMC6KO#1-1 / L_WT normalized
4F2	sp|P08195|4F2_HUMAN	Y	0.58931	0.5315
AAAT	sp|Q15758|AAAT_HUMAN	Y	0.62953	0.38724
APOC3	sp|P02656|APOC3_HUMAN	N	0.037958	0.034339
CATA	sp|P04040|CATA_HUMAN	Y	0.64265	0.49207
CD9	sp|P21926|CD9_HUMAN	Y	0.33267	0.63727
CYB5	sp|P00167|CYB5_HUMAN	Y	0.46222	0.58704
CYC	sp|P99999|CYC_HUMAN	Y	0.46059	0.58108
DDX47	sp|Q9H0S4|DDX47_HUMAN	N	0.44401	0.57464
EMC1	sp|Q8N766|EMC1_HUMAN	N	0.51103	0.41148
FADS1	sp|O60427|FADS1_HUMAN	N	0.47585	0.65086
FADS2	sp|O95864|FADS2_HUMAN	N	0.3055	0.67974
FAM3C	sp|Q92520|FAM3C_HUMAN	N	0.13015	0.2693
FKBP8	sp|Q14318|FKBP8_HUMAN	N	0.48616	0.37428
H1X	sp|Q92522|H1X_HUMAN	Y	0.23814	0.68005
H4	sp|P62805|H4_HUMAN	Y	0.15312	0.40361
KAD2	sp|P54819|KAD2_HUMAN	Y	0.25973	0.69152
LAMB3	sp|Q13751|LAMB3_HUMAN	N	0.59107	0.27541
LMNB1	sp|P20700|LMNB1_HUMAN	Y	0.61728	0.70252
LTOR1	sp|Q6IAA8|LTOR1_HUMAN	N	0.66855	0.65367
NADC	sp|Q15274|NADC_HUMAN	N	0.55962	0.56464
P5CR1	sp|P32322|P5CR1_HUMAN	Y	0.41189	0.42781
PGRC2	sp|O15173|PGRC2_HUMAN	Y	0.54622	0.63447
ROA0	sp|Q13151|ROA0_HUMAN	Y	0.29019	0.55091
ROAA	sp|Q99729|ROAA_HUMAN	Y	0.64327	0.6254
RT09	sp|P82933|RT09_HUMAN	Y	0.62019	0.62545
RT23	sp|Q9Y3D9|RT23_HUMAN	Y	0.63568	0.68099
SARNP	sp|P82979|SARNP_HUMAN	N	0.46258	0.693
SHIP1	sp|Q92835|SHIP1_HUMAN	N	0.019569	0.072773
SUMF2	sp|Q8NBJ7|SUMF2_HUMAN	Y	0.49067	0.69058
THIM	sp|P42765|THIM_HUMAN	Y	0.56182	0.70657
TMM97	sp|Q5BJF2|TMM97_HUMAN	N	0.35083	0.46437
